# Pyrophosphate homeostasis in multiple subcellular compartments is essential in *Plasmodium falciparum*

**DOI:** 10.1128/mbio.00475-26

**Published:** 2026-04-22

**Authors:** Ikechukwu Nwankwo, Hangjun Ke

**Affiliations:** 1 Center for Molecular Parasitology, Department of Microbiology and Immunology, Drexel University College of Medicine 427311, Philadelphia, Pennsylvania, USA; Iowa State University College of Veterinary Medicine, Ames, Iowa, USA

**Keywords:** *Plasmodium falciparum*, malaria, pyrophosphate, soluble pyrophosphatase, cytoplasm, mitochondrion, apicoplast

## Abstract

Pyrophosphate is a byproduct of numerous cellular reactions that use ATP or other nucleoside triphosphates to synthesize DNA, RNA, protein, and various molecules. Its degradation into monophosphate is thus crucial for the survival and proliferation of all life forms. The human malaria parasite *Plasmodium falciparum* encodes two classes of pyrophosphatases to hydrolyze pyrophosphate. The first consists of *P. falciparum* proton pumping vacuolar pyrophosphatases (e.g., PfVP1), which localize to the parasite plasma membrane and work as proton pumps. The second includes *P. falciparum* soluble pyrophosphatases (PfsPPases), which have not been well characterized. Interestingly, the gene locus of PfsPPases encodes two isoforms, PfsPPase1 (PF3D7_0316300.1) and PfsPPase2 (PF3D7_0316300.2). PfsPPase2 contains a 51-amino acid organellar localization peptide that is absent in PfsPPase1. Here, we combine reverse genetics and biochemical approaches to identify the localization of PfsPPase1 and PfsPPase2 and elucidate their individual functions. We show that PfsPPases are essential for the asexual blood stages. While PfsPPase1 solely localizes to the cytoplasm, PfsPPase2 exhibits multiple localizations, including the mitochondrion, the apicoplast, and, to a lesser extent, the cytoplasm. Our data suggest that *P. falciparum* has taken a unique evolutionary trajectory in pyrophosphate metabolism by utilizing a leader sequence to direct sPPases to multiple organelles. This differs from model eukaryotes as they generally encode multiple sPPases at distinct genetic loci to facilitate pyrophosphate degradation in cytosolic and organellar compartments. The essentiality and divergence of PfsPPases also highlight them as promising targets for the development of novel antimalarial drugs.

**IMPORTANCE:**

Malaria kills over 600,000 people annually. Understanding parasite biology is critical for identifying prospective drug targets. Malaria parasites maintain pyrophosphate (PPi) homeostasis in at least three subcellular compartments—the cytoplasm, mitochondrion, and apicoplast, where PPi is generated through various reactions. While cytoplasmic PPi is known to be degraded by soluble pyrophosphatase, it remains unclear how PPi is metabolized in the organelles of malaria parasites. Here, we discovered that *Plasmodium falciparum* encodes two soluble pyrophosphatase isoforms from a single genetic locus. The longer isoform contains an N-terminal leader sequence that targets the enzymes into the mitochondrion and the apicoplast. This dual targeting mechanism of soluble pyrophosphatases has not been previously reported in any organisms. We show that both isoforms are essential for parasite growth and development. These findings highlight the critical role of organellar PPi degradation and identify soluble pyrophosphatases as promising antimalarial drug targets.

## INTRODUCTION

Malaria threatens nearly half of the world’s population. According to a recent WHO report, about 247 million malaria cases were recorded, and ~619,000 lives were lost each year (1). The disease is caused by protozoan parasites belonging to the *Plasmodium* genus, and among the five human malaria species, *Plasmodium falciparum* is the most virulent. Despite the efforts put in place to control and eradicate the disease, progress in malaria control has been declining recently due to several factors, including the rapid emergence of drug-resistant parasites, which gradually render most antimalarial drugs ineffective (2). As a result, it is urgent to continuously investigate the parasite biology to lay the groundwork for developing novel therapeutics.

Inorganic pyrophosphatases (PPases) are essential for modulating the cellular concentrations of pyrophosphate (PPi) (3), a key metabolic byproduct of numerous cellular reactions involving the use of nucleoside triphosphates in the synthesis of DNA, RNA, proteins, and other molecules (4). They hydrolyze PPi into two monophosphates (Pi) through an irreversible reaction, which is thermodynamically favorable, resulting in the shift of the overall cellular equilibrium toward the biosynthesis of nucleic acids and/or proteins (5). The absence of PPases would result in the accumulation of PPi to toxic levels, affecting numerous biological processes in the cells; therefore, PPases are essential in all organisms. In *Caenorhabditis elegans*, null mutations of its PPases led to developmental arrest at the larval stage as evidenced by defects in the animal’s intestine (6). Mutations of PPases in the budding yeast (*Saccharomyces cerevisiae*) also cause cell cycle arrest and cell death (7). Furthermore, various studies on pathogenic organisms have focused on PPases as potential drug targets. For example, the drug-resistant strains of *Staphylococcus aureus* are highly susceptible to small molecule inhibitors against PPases (8).

Unlike fungi and metazoans that only express soluble pyrophosphatases, *P. falciparum* encodes two distinct kinds of inorganic pyrophosphatases, including the soluble pyrophosphatase (*P. falciparum* soluble pyrophosphatase, PfsPPase) and the membrane-bound H^+^-translocating vacuolar pyrophosphatases (*P. falciparum* vacuolar pyrophosphatases, PfVP1 and PfVP2) (9). Recently, we have demonstrated that PfVP1 is uniquely localized to the parasite plasma membrane (10), showing a sharp contrast to other single-cellular parasites such as *Toxoplasma gondii* and Kinetoplastids whose VP1 proteins localize to intracellular organelles called acidocalcisomes (11–14). We further demonstrated that ring stage parasites utilize PfVP1 to maintain the plasma membrane potential, cytosolic pH, and energy homeostasis (10).

**Fig 1:**
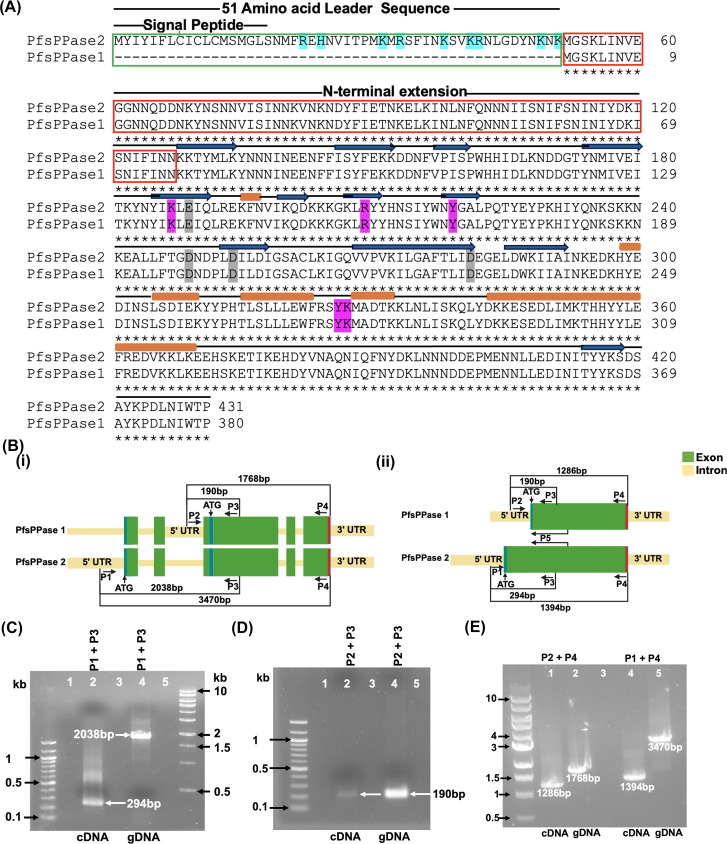
The gene locus of PfsPPases. (**A**) Protein sequence alignment of PfsPPase1 and PfsPPase2. The 51-aa leader sequence in PfsPPase2 is highlighted in a green box. The N-terminal extension of PfsPPases is shown in a red box. PfsPPases are featured with beta strands (dark blue arrows) and alpha helices (brown rectangular bars). Some critical amino acids are shaded in magenta (involved in pyrophosphate binding) or gray (involved in metal binding). This figure is modified from the previous study (18). (**B**) Schematic representation of the PfsPPase genetic locus as depicted in PlasmoDB (www.plasmodb.org). (i) with introns representing the genomic DNA (gDNA), (ii) without introns representing PfsPPase1 and 2 transcripts. (**C**) Gel electrophoresis of PfsPPase2 amplified from DNase-treated complementary DNA (cDNA) and gDNA by primers P1 + P3. (**D**) Gel electrophoresis of PfsPPase1 amplified from DNase-treated cDNA and gDNA by primers P2 + P3. (**E**) The full-length transcripts of both PfsPPase2 and PfsPPase1 were amplified from cDNA using P1 + P4 or P2 + P4, respectively.

On the other hand, the functions of *P. falciparum* soluble pyrophosphatases (PfsPPases) remain poorly characterized. Interestingly, the genetic locus of PfsPPases (PF3D7_0316300), as depicted in PlasmoDB (www.plasmoDB.org), potentially encodes two isoforms: PF3D7_0316300.1 (380 aa) and PF3D7_0316300.2 (431 aa), which we henceforth refer to as PfsPPase1 and PfsPPase2, respectively. Both enzymes have the same peptide sequence except that PfsPPase2 contains a putative targeting signal (51 aa) at its N-terminus (Fig. 1A). The 51-aa leader sequence contains a signal peptide predicted by SignalP 4.0 and several positively charged and hydrophobic amino acid residues (Fig. 1A), which resemble the features of organellar targeting sequences (15–17). Furthermore, MitoProt predicts PfsPPase2’s mitochondrial localization with a high confidence score of 0.8, suggesting that PfsPPases are not only cytosolic but may also be organellar. However, this expected organellar localization of PfsPPases was not reported in the previous study, which solely focused on one isoform (PfsPPase1) but not two (18). Hence, it remains unclear why malaria parasites potentially need two soluble pyrophosphatases.

Derived from secondary endosymbiosis, *Plasmodium* parasites possess three genomes in the nucleus (19, 20), the mitochondrion (21–23), and the apicoplast (a plastid derivative) (24–27), which all need mechanisms of PPi degradation to avoid toxicity. While PPi generated in the nucleus can be trafficked/diffused through the nuclear pores into the cytoplasm and degraded by cytoplasmic sPPases as previously suggested (28), it remains unknown how the mitochondrion and the apicoplast deal with PPi homeostasis in malaria parasites. In this study, we demonstrate that PfsPPase1 localizes to the parasite cytoplasm, whereas PfsPPase2 utilizes its 51-aa leader sequence to reach the mitochondrion and the apicoplast. Although PfsPPases have previously been predicted to be mutable in *P. falciparum* by a piggyBac mutagenesis study (29), we reveal that both isoforms are essential in the asexual blood stages. To the best of our knowledge, this is the first study to demonstrate that *Plasmodium falciparum* employs a non-canonical strategy to maintain PPi homeostasis in endosymbiotic organelles, as other eukaryotes often encode organellar-specific sPPases that share low sequence identity with cytoplasmic counterparts. Furthermore, we speculate that chemical inhibition of soluble pyrophosphatases, if available, could exert antimalarial effects across three major parasite compartments, including the cytoplasm, the mitochondrion, and the apicoplast.

## RESULTS

### The genetic locus of soluble pyrophosphatases in *P. falciparum* encodes two isoforms

The gene locus of PfsPPases (PF3D7_0316300) as observed on PlasmoDB potentially encodes two isoforms: PfsPPase1 (PF3D7_0316300.1) and PfsPPase2 (PF3D7_0316300.2). Their genetic structures are schematically depicted in Fig. 1B, with introns present (genomic DNA) or spliced out (complementary DNA [cDNA]). PfsPPase1 and PfsPPase2 have the same protein sequence except that the latter contains an N-terminal 51-aa leader peptide (Fig. 1A), which indicates a possible organellar localization. RNA-seq data available on PlasmoDB further support that the two short exons upstream within the gene locus of PfsPPase2 are transcribed despite their long distance to the next exon (approx. 1,400 bp) (Fig. S1). Since PfsPPase1 possesses the same protein sequence as PfsPPase2 except the leader peptide, we reasoned that PfsPPase1 is either independently transcribed from the genetic locus or a product of alternative splicing from the PfsPPase2 transcript. To differentiate these two possibilities, we designed primers to amplify several regions of the PfsPPase gene locus using DNase-treated complementary DNA and genomic DNA (gDNA) as the template (Fig. 1B). PCR confirmed that PfsPPase2 is transcribed as expected using primers P1 and P3 (Fig. 1C) as the sizes of amplicons match the annotated gene model in PlasmoDB. On the other hand, PfsPPase1 is independently transcribed as primers P2 and P3 amplified the same product (190 bp) from either DNase-treated cDNA or gDNA (Fig. 1D). If PfsPPase1 were a spliced product of PfsPPase2, the 190 bp amplicon would not be amplified from cDNA with primers P2 and P3 because the P2 binding region should be spliced out upon intron removal. To further confirm that PfsPPase1 is independently transcribed, we verified the full-length transcripts of the two isoforms using RT-PCR (Fig. 1E) and sequenced each transcript using Sanger sequencing. The sequencing data and sequence alignment revealed that PfsPPase1 and PfsPPase2 possess separate 5′ untranslated regions (UTRs) (Fig. S2). Therefore, PfsPPase1 is an independent transcript, not a product of alternate splicing from the PfsPPase2 transcript. Knowing that PfsPPase1 is independently transcribed, we speculated that the 1.4 kb intron upstream of PfsPPase1 should work as the promoter. To test this hypothesis, we amplified the 1.4 kb sequence, cloned it into a plasmid to drive the expression of GFP (fused to yeast dihydroorotate dehydrogenase [yDHOD]) (30), and generated a parasite line, NF54attB-1.4kb-yDHOD-GFP. Live-cell microscopy showed robust expression of GFP in the parasite cytosol, suggesting that the 1.4 kb intron works as a promoter (Fig. S3).

Altogether, these results strongly suggest that the genetic locus of PF3D7_0316300 encodes two soluble pyrophosphatases, PfsPPase1 and PfsPPase2. The two isoforms are driven by different promoters and are independently transcribed. PfsPPase2 contains the 51-aa leader sequence, but its remaining peptide sequence is identical to that of PfsPPase1.

### PfsPPases are essential for parasite development and growth

**Fig 2:**
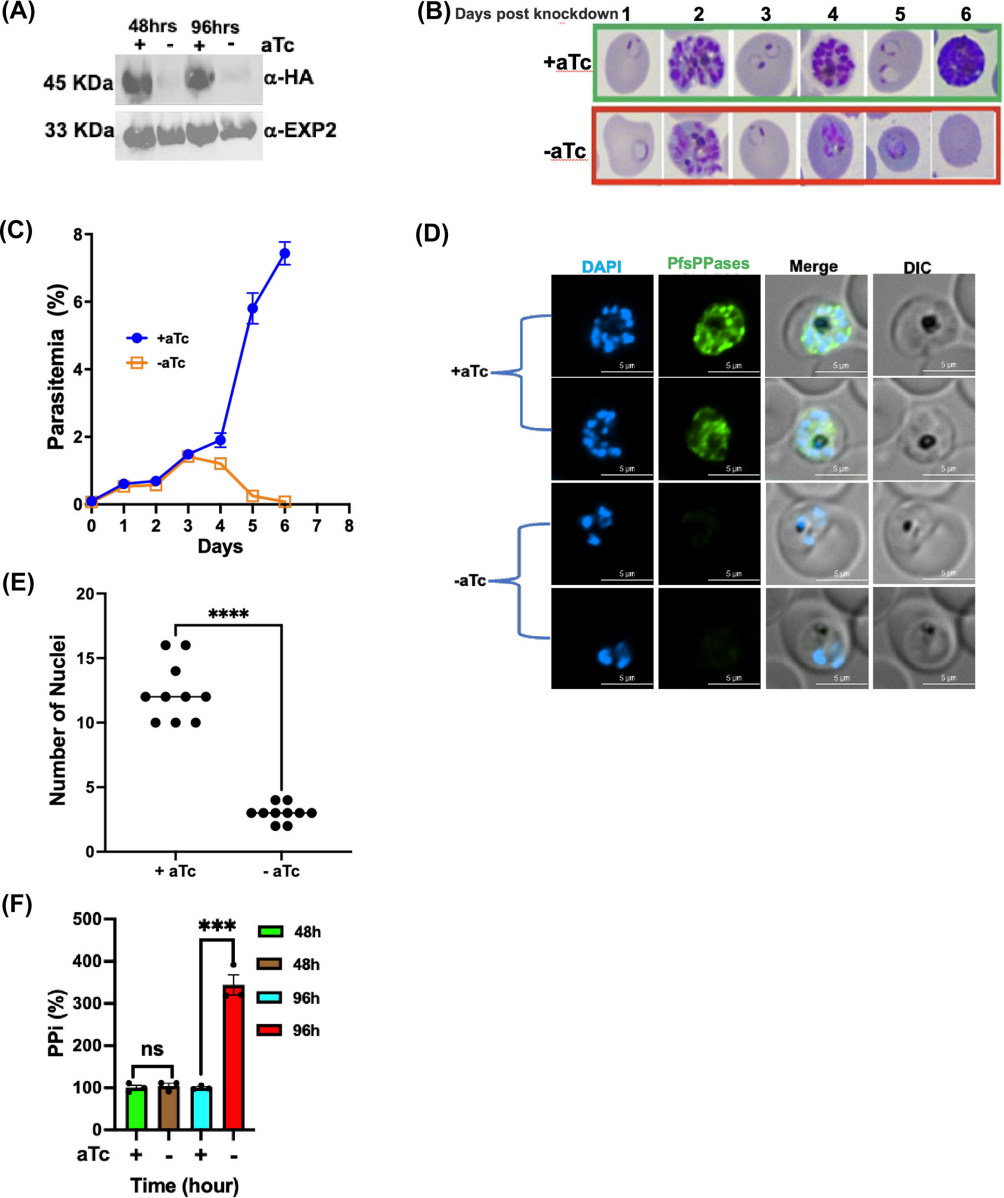
PfsPPases are crucial for parasite development and growth. (**A**) Western blot confirming the knockdown of PfsPPases-3HA upon aTc removal in the transgenic parasite line, NF54attB-PfsPPases-3HA^APT^. The blot was probed with anti-HA and re-probed with anti-PfEXP2 to show loading controls. (**B**) Giemsa-stained thin blood smears showing parasite morphological changes after aTc removal for 6 days (three intraerythrocytic developmental cycles). Green box, aTc (+). Red box, aTc (−). (**C**) Parasitemia of the knockdown experiment over the course of 6 days. Parasitemia was determined by light microscopy in triplicate samples. (**D**) Visualization of parasite DNA stained by DAPI post-knockdown of PfsPPases for 96 h. Green, PfsPPases-3HA; scale bars, 5 μm. (**E**) Number of nuclei in parasites post knockdown of PfsPPases for 96 h. (**F**) Measurement of PPi in the PfsPPase knockdown parasites after aTc removal for 48 and 96 h. The concentration of PPi in each condition was measured using the PPi sensor from Sigma and normalized to total protein quantities in each sample, as described previously (10). The relative PPi concentration of aTc (−) was compared to the aTc (+) control at each time point. Statistical analysis was done by *t*-tests. ***, *P* value (0.006); ns, non-significant. Experiments of panels A–D have been repeated more than four times; panel F was repeated twice.

To directly examine the essentiality of PfsPPases in the parasite, we employed CRISPR/Cas9 (31, 32) and generated a conditional knockdown line in NF54attB (33) using the TetR-DOZI-aptamer system (34, 35) (Fig. S4A). The parasite line was named NF54attB-PfsPPases-3HA^APT^, in which the gene locus of PfsPPases was endogenously tagged with a triple hemagglutinin (3HA) tag at the C-terminus. The genotype of NF54attB-PfsPPases-3HA^APT^ was confirmed by PCR (Fig. S4B). The parasites were cultured in media supplemented with 250 nM of anhydrotetracycline (aTc) to sustain PfsPPase expression (Fig. S4C through E). Next, we tightly synchronized the culture, initiated knockdown by aTc removal in Percoll-isolated schizont stage parasites, and examined parasite development over several intraerythrocytic developmental cycles (IDCs). After aTc was removed, we confirmed the reduction of the endogenously 3HA-tagged PfsPPase proteins at 48 and 96 h post-knockdown (Fig. 2A; Fig. S5A). Our C-terminal tagging strategy should theoretically produce two tagged proteins with noticeable differences in molecular weights, 45 kDa (PfsPPase1-3HA, 380 aa plus tag) versus 51 kDa (PfsPPase2-3HA, 431 aa plus tag). However, Western blot only detected one band near the anticipated molecular weight of PfsPPase1 (45 kDa), suggesting that the PfsPPase2’s leader peptide was efficiently removed, and no precursor protein of PfsPPase2 (51 kDa) was detectable. This phenomenon is not uncommon in *P. falciparum* as mitochondrially localized proteins often exhibit the processed form with no precursors detectable in Western blot (36, 37). Upon the knockdown of PfsPPases, Giemsa-stained images showed that although PfsPPase-depleted parasites successfully finished the first IDC, they were completely arrested in the trophozoite stage of the second IDC and failed to develop further into schizonts, resulting in a severe block in parasite replication and subsequent lysis of the parasites in the third IDC (Day 6) (Fig. 2B). We quantified parasitemia of both the control and knockdown cultures and showed that PfsPPases were essential (Fig. 2C). We further investigated the PfsPPase knockdown parasites under a fluorescence microscope on Day 4 post-aTc removal when parasite development was arrested (Fig. 2D). In agreement with Western blot data in Fig. 2A, the PfsPPase proteins were undetectable at 96 h post-aTc removal. While the control parasites had already progressed into the schizont stage with an average of 12 nuclei, the PfsPPases knockdown parasites remained stalled/arrested at the late trophozoite stage (or an early schizont stage), exhibiting only two or three nuclei (Fig. 2D and E). To investigate whether the developmental arrest observed upon PfsPPases knockdown is due to the accumulation of PPi, we quantified PPi levels using a kit (Materials and Methods). At 48 h of aTc removal, we observed no significant differences in PPi levels between the control and knockdown parasites. However, at 96 h of knockdown, there was a significant increase in PPi levels in the PfsPPases-depleted parasites (Fig. 2F). Altogether, these findings demonstrate that knockdown of PfsPPases causes severe development arrest, elevated PPi levels, and parasite death, validating the essentiality of PfsPPases for *P. falciparum* in the asexual blood stages.

### Function of the two PfsPPase isoforms: partial or full rescue of the PfsPPases knockdown parasites by PfsPPase1 or PfPPase2, respectively

Since our knockdown strategy affects both PfsPPase isoforms simultaneously, the observed phenotypes in Fig. 2 represent the gross defects caused by the loss of both PfsPPase1 and PfsPPase2. To independently characterize the two isoforms and determine their individual functions, we generated two new parasite lines in the available NF54attB-PfsPPases-3HA^APT^ line. These lines were named as NF54attB-PfsPPases-3HA^APT^-PfsPPase1-3Myc (PfsPPase1-3Myc in short) and NF54attB-PfsPPases-3HA^APT^-PfsPPase2-3Myc (PfsPPase2-3Myc in short), which contain the endogenous PfsPPases tagged with 3HA and aptamers and a constitutively expressed episomal copy of PfsPPase1 or PfsPPase2 tagged with 3Myc. The episomal copy was expressed under the mitoribosomal protein L2 (mRL2) promoter, which has been routinely used to drive protein expression at moderate levels (37, 38). These two lines, PfsPPase1-3Myc and PfsPPase2-3Myc, allowed us to investigate the function of each isoform after conditional knockdown of the endogenous 3HA-tagged PfsPPases by aTc removal.

**Fig 3:**
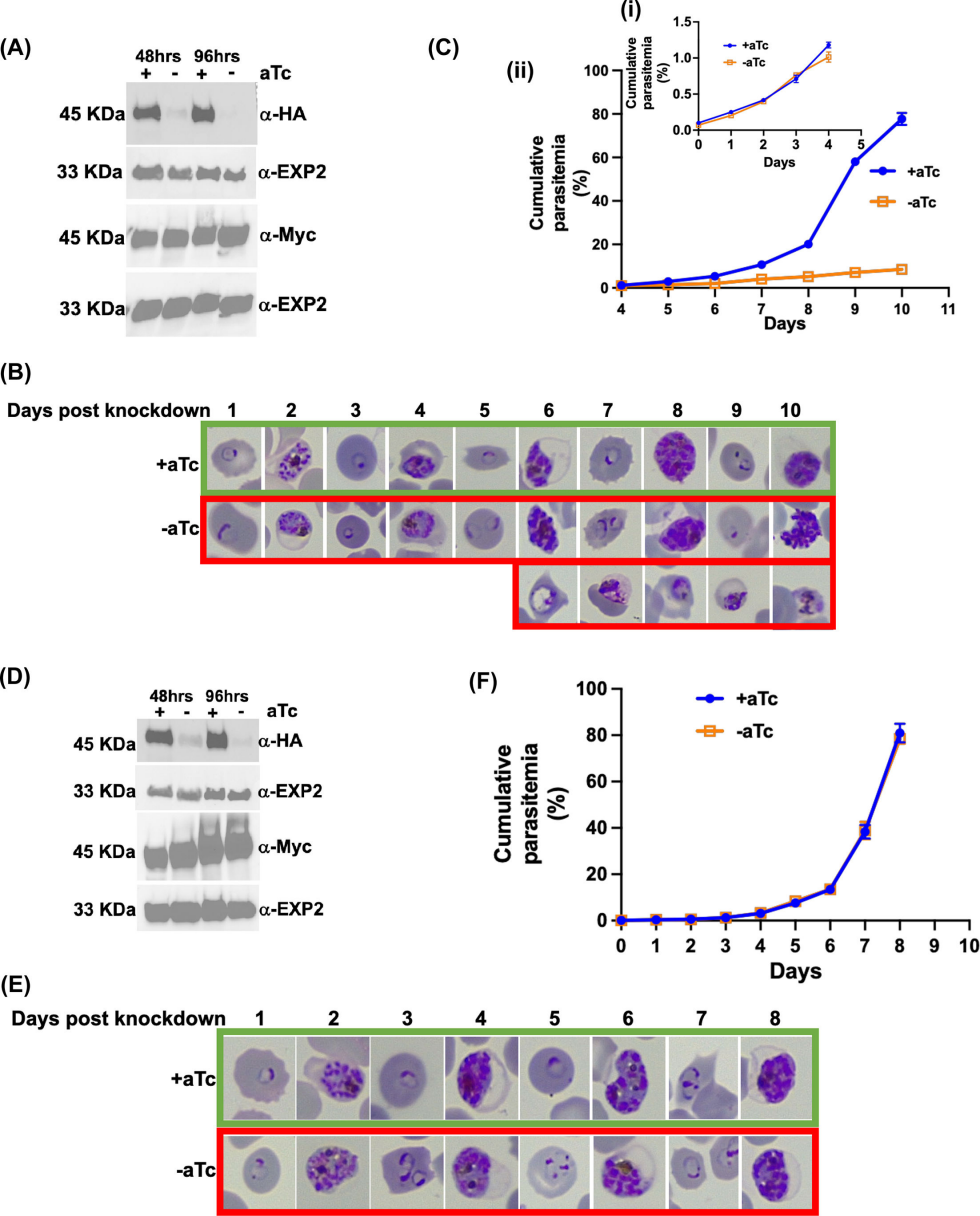
PfsPPase1 partially complements PfsPPases knockdown parasites, while PfsPPase2 fully complements them. (**A**) Western blot analysis of protein lysate from NF54attB-PfsPPase-3HA^APT^-PfsPPase1-3Myc. The blot was probed with anti-HA to verify knockdown of the endogenously tagged PfsPPases-3HA and re-probed with anti-PfEXP2 to show loading controls. Duplicate samples were also blotted with anti-Myc to confirm the expression of the episomally tagged PfsPPase1-3Myc and re-probed with anti-PfEXP2 to show loading controls. (**B**) Giemsa-stained thin blood smears showing parasite morphological changes over 10 days after aTc removal. Green box, aTc (+). Red box, aTc (−). (**C**) (i) Parasitemia for the first 4 days of the knockdown experiment. (ii) Parasitemia for the last 6 days. Parasitemia was determined by microscopic counting in at least three biological replicates. These experiments were repeated at least three times. (**D**) Western blot analysis of protein lysate from NF54attB-PfsPPase-3HA^APT^-PfsPPase2-3Myc. The blot was probed with anti-HA to verify knockdown of the endogenously tagged PfsPPases-3HA and re-probed with anti-PfEXP2 to show loading controls. Duplicate samples were also blotted with anti-Myc to confirm the expression of the episomally tagged PfsPPase2-3Myc and re-probed with anti-PfEXP2 to show loading controls. (**E**) Giemsa-stained thin blood smears showing parasite morphological changes after aTc removal. Green box, aTc (+). Red box, aTc (−). (**F**) Parasitemia of the knockdown experiment after the removal of aTc. Parasitemia was determined by microscopic counting in triplicate samples. These experiments were repeated four times.

In the PfsPPase1-3Myc parasite line, we tightly synchronized the culture, removed aTc, and analyzed the protein levels of the endogenous PfsPPases-3HA and the episomal PfsPPase1-3Myc. Western blot confirmed the constitutive expression of the episomal PfsPPase1-3Myc, while the endogenous 3HA-tagged PfsPPases were efficiently silenced upon aTc removal for 48 and 96 h, suggesting that parasites solely relied on PfsPPase1-3Myc for PPi hydrolysis in the knockdown conditions (Fig. 3A; Fig. S5B and C). To observe the effects of PfsPPase1-3Myc alone on parasite development, we repeated the knockdown experiment and extended the aTc ± cultures for 10 days (five IDCs). In contrast to the control culture (aTc +), which progressed normally through the IDC, we observed that the PfsPPase1-3Myc alone culture (aTc −) grew fine on Day 4, however, it started to show growth defects in the third IDC after aTc removal (Fig. 3B). On Day 6, about 40% of the PfsPPase1-3Myc alone culture exhibited abnormal parasites, with their morphologies resembling arrested trophozoites undergoing lysis. This abnormal phenotype increased in frequency in the fourth and fifth IDCs post-aTc removal, suggesting that a significant portion of parasites was arrested in the trophozoite stage and unable to develop further into the schizont stage. Quantification of parasitemia over the 10-day knockdown course confirmed this developmental arrest (Fig. 3C). Altogether, these findings show that complementation with PfsPPase1 alone in the PfsPPases knockdown parasites rescues parasite growth in the second cycle post aTc removal, but it is insufficient to fully rescue in long-term, suggesting that PfsPPase1 is essential but parasites likely need both isoforms.

Using similar approaches, in the PfsPPase2-3Myc parasite line, we assessed the effects of PfsPPase2 alone on parasite development by knocking down the endogenously 3HA-tagged PfsPPases. Again, Western blot confirmed that PfsPPase2-3Myc was expressed independently of aTc, while the endogenous 3HA-tagged PfsPPases were effectively knocked down upon aTc removal for 48 or 96 h (Fig. 3D; Fig. S5D and E). In contrast to PfsPPase1 that insufficiently rescued the loss of endogenous PfsPPases, PfsPPase2 alone completely sustained parasite development without noticeable defects in parasite morphologies or numbers over four IDCs (Fig. 3E and F). These results suggest that PfsPPase2 is essentail and plays some functions that are not fulfilled by PfsPPase1 alone, indicating that the parasites need both PfsPPases.

**Fig 4:**
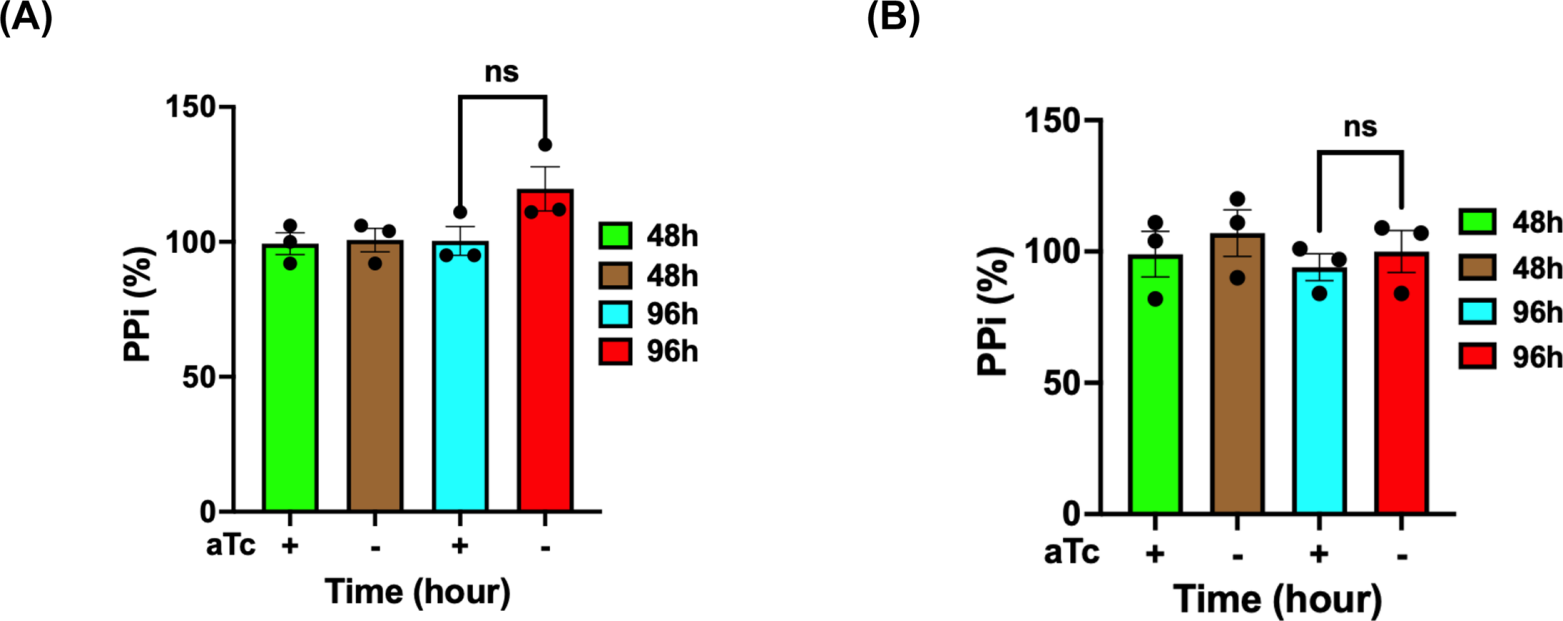
PPi levels in PfsPPase1- and PfsPPase2-complemented lines. Measurement of PPi in the PfsPPase1-3myc and PfsPPase2-3myc parasite lines after aTc removal for 48 and 96 h from the schizont stage. PPi concentration was measured by a Sigma kit and quantified in nanomoles of PPi/mg of protein, as described previously (10). The relative PPi concentration at various time points was normalized to that of the control (aTc +). (**A**) PfsPPase1-3myc. (**B**) PfsPPase2-3myc. Mean ± SEM of the three measurements is shown, and statistical analysis was done by *t*-test. ns, not significant; *P* values (0.1186 and 0.5628, respectively). These experiments were repeated three times.

To further verify whether PfsPPase1-3Myc and PfsPPase2-3Myc worked enzymatically properly in degrading PPi, we quantified PPi levels in the parasite lines, PfsPPase1-3Myc and PfsPPase2-3Myc, grown in aTc (±) conditions at different time points upon aTc removal. As shown in Fig. 2F, the parental line NF54attB-PfsPPases-3HA^APT^ had elevated PPi levels at 96 h post-aTc removal. However, at the same time point, the elevated PPi levels in the aTc (−) conditions were completely abolished when either PfsPPase1-3Myc or PfsPPase2-3Myc was expressed (Fig. 4). This indicates that the episomal PfsPPase1-3Myc or PfsPPase2-3Myc was sufficient to prevent PPi accumulation when the endogenous PfsPPases were ablated. We extended our PPi measurements to 192 h (four IDCs) of aTc removal and still observed no significant differences between the aTc (±) groups (Fig. S6), suggesting that the episomal PfsPPases work in the long term. However, due to the lack of purification protocols for isolating parasite organelles at high purity, we were unable to differentiate PPi levels in various subcellular compartments (see Discussion). Altogether, these results indicate that both PfsPPase1 and PfsPPase2 are enzymatically functioning in the parasites to prevent PPi accumulation.

### Subcellular localization of PfsPPases by fixed- or live-cell fluorescence microscopy

Our results suggested that while PfsPPase1 partially rescues the loss of the endogenous PfsPPases, PfsPPase2 fully rescues. To verify their subcellular localization, we utilized both fixed- and live-cell fluorescence microscopy in multiple transgenic parasite lines.

**Fig 5:**
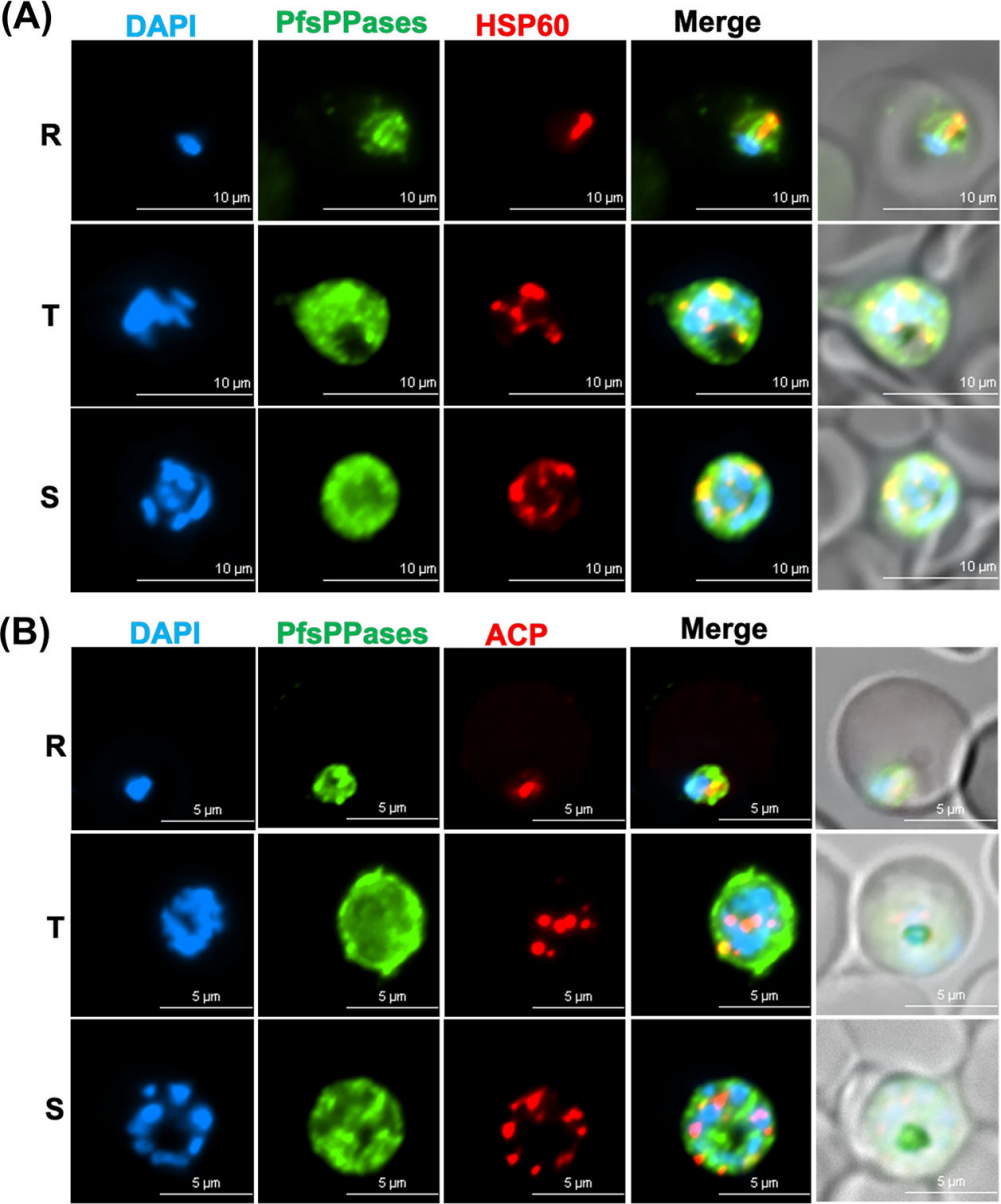
Subcellular localization of PfsPPases. (**A and B**) Immunofluorescence assay demonstrating the expression and localization of PfsPPases-3HA throughout the parasite’s IDC. R, ring; T, trophozoite; and S, schizont. DAPI stained the nuclei (blue), and PfsPPases-3HA is shown in green. (**A**) Colocalization of PfsPPases with the mitochondrion detected by anti-PfHSP60 (red). Scale bar, 10 µm. The Pearson correlation coefficient between green and red fluorescence was calculated from *n* = 10 parasites at each stage. Ring (0.7713 ± 0.0548), trophozoite (0.7197 ± 0.0845), and schizont (0.7883 ± 0.0374). (**B**) Colocalization of PfsPPases with the apicoplast detected by anti-PfACP (red). Scale bar, 5 µm. The Pearson correlation coefficient between green and red fluorescence was calculated from *n* = 10 parasites at each stage. Ring (0.6493 ± 0.0632), trophozoite (0.5979 ± 0.0781), and schizont (0.5967 ± 0.0943).

First, in the NF54attB-PfsPPases-3HA^APT^ line, we conducted an immunofluorescence assay (IFA) using antibodies against HA, PfHSP60 (heat shock protein 60, a mitochondrial marker [39, 40]), and PfACP (acyl carrier protein, an apicoplast marker [27, 41]). Our data showed that PfsPPases exhibit strong diffused staining throughout the parasite, indicating that PfsPPases are predominantly localized to the parasite cytosol, in agreement with the previous study (18). However, IFA also revealed that PfsPPases display some punctate staining patterns, which partially co-localize with the structures detected by anti-PfHSP60 (Fig. 5A) or anti-PfACP antibodies (Fig. 5B). This result suggests that endogenous PfsPPases mainly localize to the parasite cytoplasm and additionally to the organelles (the mitochondrion and the apicoplast).

**Fig 6:**
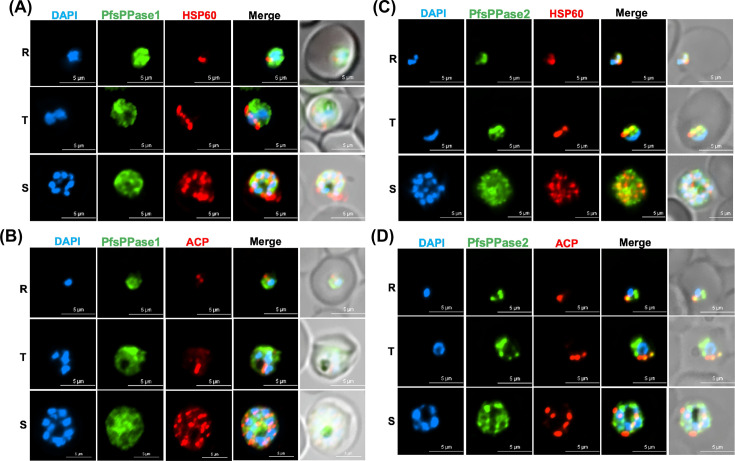
Subcellular localization of PfsPPase1 and PfsPPase2. Immunofluorescence assays of NF54attB-PfsPPase-3HA^APT^-PfsPPase1-3Myc (**A and B**) or NF54attB-PfsPPase-3HA^APT^-PfsPPase2-3Myc (**C and D**), conducted at 96 h post-aTc removal to deplete the endogenous PfsPPases. The samples were verified by anti-Myc and anti-PfHsp60 (**A and C**) or by anti-Myc and anti-PfACP (**B and D**). PfHSp60 is a mitochondrial marker; PfACP is an apicoplast marker. Scale bars, 5 μm. In panels **A–D**, Pearson correlation coefficient of green and red fluorescence was derived from *n* = 10 parasites of each stage. The values are as follows: (**A**) ring (0.4791 ± 0.1082), trophozoite (0.4591 ± 0.0873), and schizont (0.4656 ± 0.0744); (**B**) ring (0.3977 ± 0.0632), trophozoite (0.4132 ± 0.0612), and schizont (0.4483 ± 0.0552); (**C**) ring (0.7729 ± 0.0429), trophozoite (0.7532 ± 0.0537), and schizont (0.7475 ± 0.0643); and (**D**) ring (0.6834 ± 0.0496), trophozoite (0.6753 ± 0.0590), and schizont (0.6190 ± 0.0602).

Second, we performed IFA in PfsPPase1-3Myc or PfsPPase2-3Myc lines that expressed only one isoform after the endogenous 3HA-tagged PfsPPases were depleted by aTc removal for 96 h. IFA revealed that PfsPPase1 was mainly localized to the parasite cytosol with no apparent colocalization with markers against the mitochondrion or the apicoplast (Fig. 6A and B). By contrast, PfsPPase2 exhibited strong colocalization with the mitochondrion and partial colocalization with the apicoplast (Fig. 6C and D). Unexpectedly, PfsPPase2 was not merely organellar; it was also detected in the parasite cytosol, as IFA showed some diffused staining besides the punctate structures. These results indicate that while PfsPPase1 is solely cytoplasmic, PfsPPase2 localizes to multiple compartments, including the mitochondrion, the apicoplast, and the cytoplasm. While the mitochondrial localization of PfsPPase2 corroborates well with the MitoProt prediction (a score of 0.8), the mechanisms by which PfsPPase2 localizes to the apicoplast and cytoplasm remain unknown (see Discussion). Nevertheless, the localization patterns of PfsPPases are consistent with their functions: PfsPPase2 is fully sufficient to maintain PPi homeostasis of the entire parasite, whereas PfsPPase1 is mainly responsible for the cytoplasm.

Third, to further verify the organellar localization of PfsPPase2, we generated another parasite line (NF54attB-PfsPPase2^leader^-mNeonGreen) in which the 51-aa leader sequence of PfsPPase2 was fused with mNeonGreen (mNG), and the fusion gene was integrated into a non-essential genomic locus (42) and expressed under the mRL2 promoter (Fig. S7A). Using this parasite line in live-cell microscopy, we stained it with MitoTracker and confirmed that the leader sequence of PfsPPase2 is sufficient to direct the protein to the mitochondrion (Fig. S7B). Again, this result corroborates well with the high predicted score of PfsPPase2 being mitochondrial (0.8 by MitoProt). Moreover, using IFA with anti-PfACP antibody, we showed that the leader sequence is also capable of guiding mNG into the apicoplast (Fig. S7B). Thus, it appears that the 51-aa leader sequence is sufficient to guide PfsPPase2 to the organelles, including the mitochondrion and the apicoplast.

**Fig 7:**
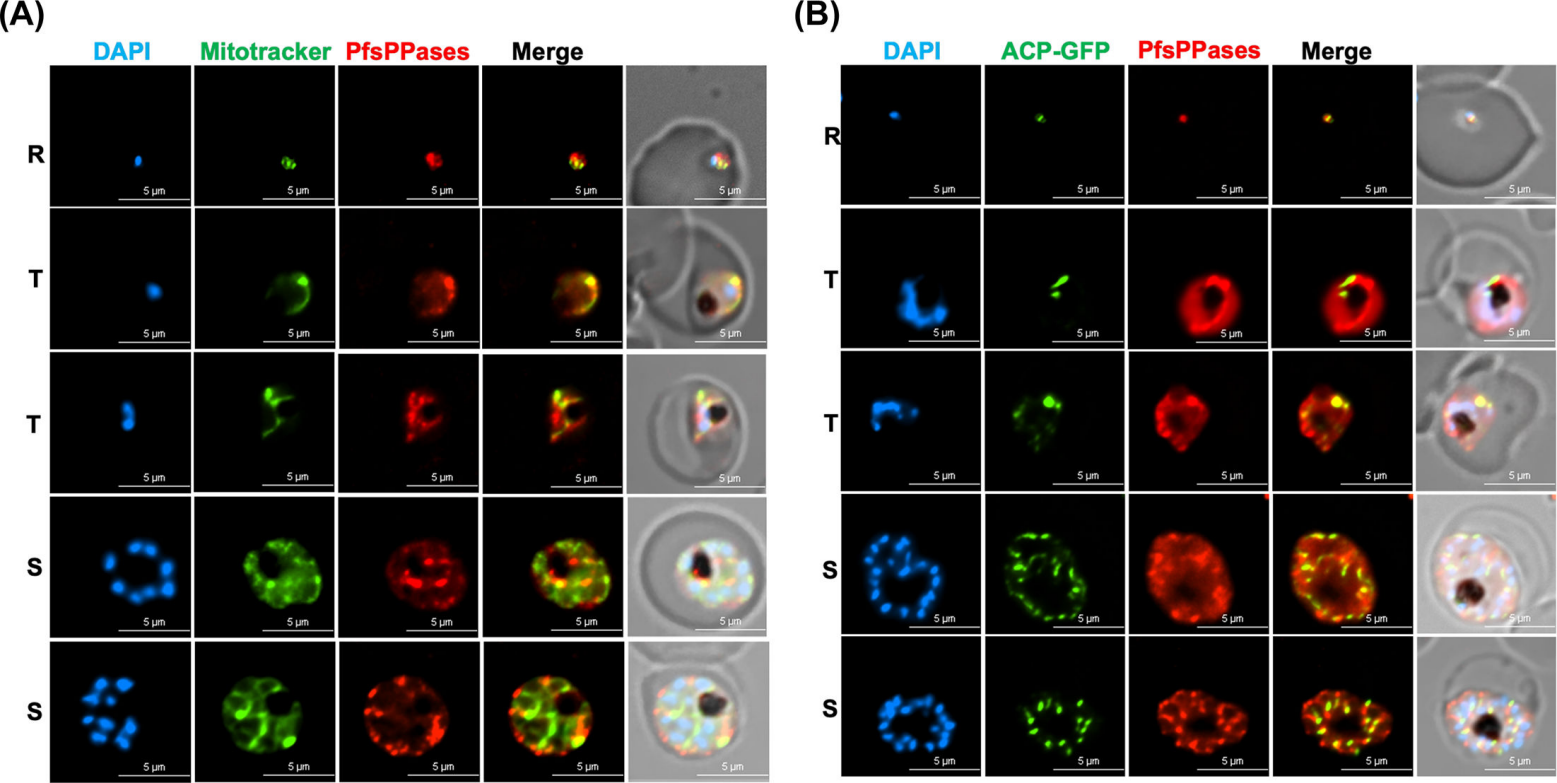
Subcellular localization of PfsPPases via live microscopy. Live imaging of NF54attB-PfsPPases-mRuby^APT^ and PfMEV-PfsPPases-mRuby^APT^ showing expression and localization of the enzymes throughout the parasite’s IDC. R, ring; T, trophozoite; and S, schizont. The nuclei were stained with DAPI. Red, PfsPPases-mRuby. (**A**) Colocalization of PfsPPases with the mitochondrion detected by green MitoTracker using NF54attB-PfsPPases-mRuby^APT^ parasites. Scale bars, 5 μm. Pearson correlation coefficient of green and red fluorescence was derived from *n* = 10 parasites of each stage. Ring (0.8967 ± 0.0489), trophozoite (0.8252 ± 0.0432), and schizont (0.8303 ± 0.0563). (**B**) Colocalization of PfsPPases with the apicoplast labeled by SFG (guided by the first 55 aa of acyl carrier protein) using PfMEV-PfsPPases-mRuby^APT^ parasites. Scale bars, 5 μm. Pearson correlation coefficient of green and red fluorescence was derived from *n* = 10 parasites of each stage. Ring (0.8609 ± 0.0664), trophozoite (0.8599 ± 0.0833), and schizont (0.8000 ± 0.0766).

Finally, to validate the localization data as shown above, we generated two new parasite lines that endogenously tagged PfsPPases with mRuby for live-cell microscopy in the wild-type and PfMev background. PfMev was generously provided by Dr. Sean Prigge (Johns Hopkins University), and it contains a Super Folder GFP (SFG)-labeled apicoplast (43). The parasite lines were named NF54attB-PfsPPases-mRuby^APT^ and PfMev-PfsPPases-mRuby^APT^. Using the NF54attB-PfsPPases-mRuby^APT^ parasite line, we stained the parasite’s mitochondrion with green MitoTracker and observed strong fluorescence of PfsPPases in the mitochondrion and cytosol across all stages of the parasite’s IDC, confirming the localization of PfsPPases to both the cytosol and mitochondrion (Fig. 7A; additional images in Fig. S8A). Interestingly, in the PfMev-PfsPPases-mRuby^APT^ parasite line, live-cell microscopy detected fluorescence of PfsPPases in the cytoplasm as well as the apicoplast across different stages of the parasite’s IDC (Fig. 7B; additional images in Fig. S8B). Collectively, we have conducted localization studies in six transgenic parasite lines, and the data reveal that PfsPPases are localized to the cytosol, the mitochondrion, and the apicoplast.

### Subcellular localization of PfsPPases by a cytosolic protein leakage assay

**Fig 8:**
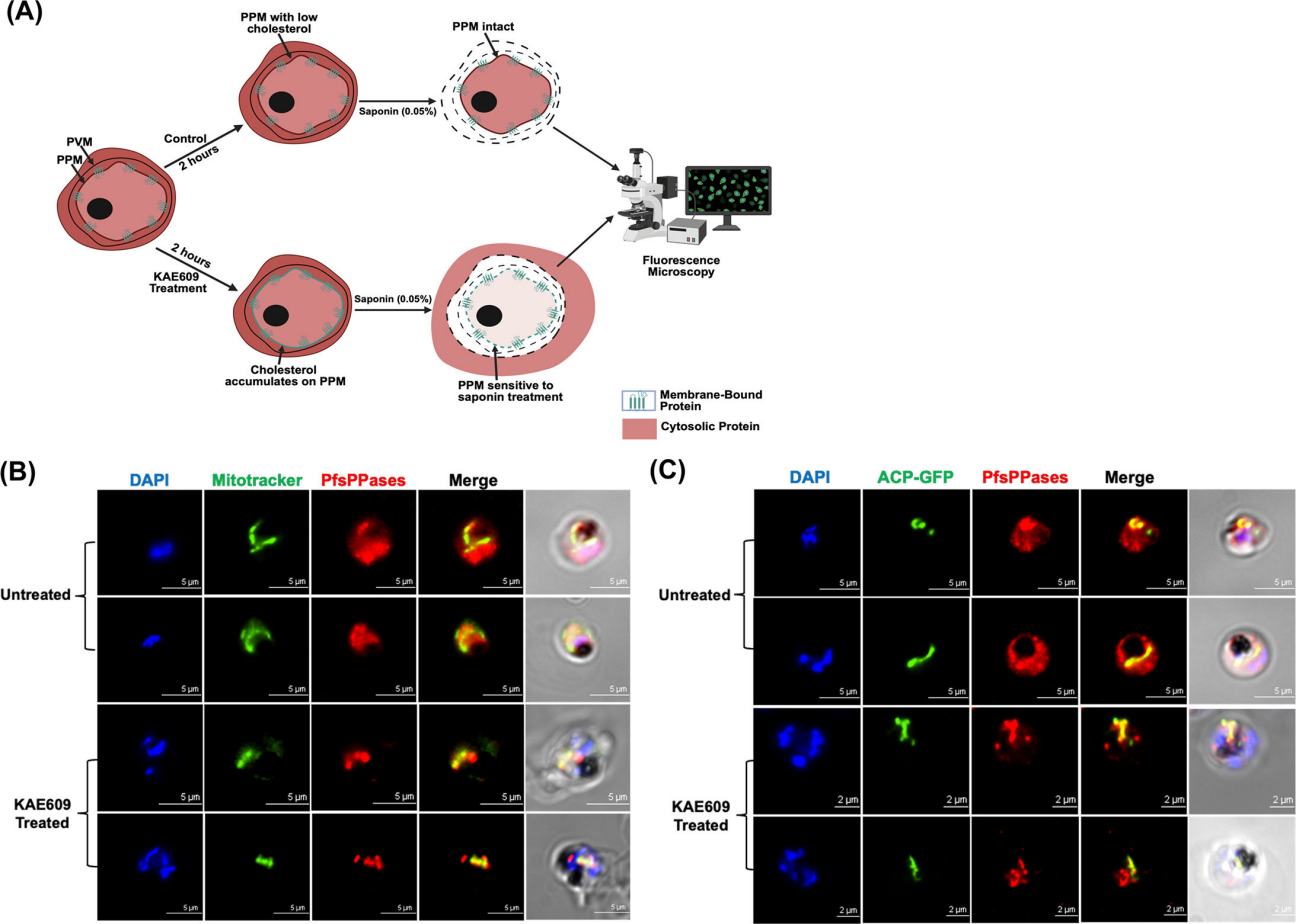
Confirming subcellular localization of PfsPPases using a cytosolic leakage assay. (**A**) Schematic diagram illustrating the cytosolic protein leakage assay for live-cell microscopy. The NF54attB-PfsPPases-mRuby^APT^ and PfMEV-PfsPPases-mRuby^APT^ parasites were treated with KAE609 (a PfAPT4 inhibitor) for 2 h, followed by saponin treatment. The saponin-treated parasites were observed by live-cell microscopy. (**B**) Colocalization of PfsPPases and the mitochondrion in the NF54attB-PfsPPases-mRuby^APT^ line after the leakage of cytoplasmic proteins. The mitochondrion was pre-stained with green MitoTracker. Pearson correlation coefficient of green and red fluorescence was derived from *n* = 10 parasites in the KAE609 untreated sample (0.8241 ± 0.0680) or treated sample (0.9095 ± 0.0980). Scale bars, 5 μm. (**C**) Colocalization of PfsPPases and the apicoplast labeled by SFG (guided by the first 55 aa of acyl carrier protein) in the PfMEV-PfsPPases-mRuby^APT^ line. Pearson correlation coefficient of green and red fluorescence was derived from *n* = 10 parasites in the KAE609 untreated sample (0.8666 ± 0.0540) or the treated sample (0.8999 ± 0.0623). Scale bars, 5 μm.

As demonstrated above (Fig. 5 to 7; Fig. S7 and S8), we have utilized both fixed- and live-cell microscopy to verify the subcellular localization of PfsPPases. Our results reveal that while PfsPPase1 is predominantly cytoplasmic, PfsPPase2 exhibits a more diverse distribution, localizing to the mitochondrion, apicoplast, and cytoplasm. To further verify this complex localization pattern of PfsPPases, we utilized a new assay, originally developed by Dr. Akhil Vaidya’s group (Drexel University). This assay is termed “cytosolic protein leakage assay,” and the schematic is presented in Fig. 8A. As published previously by Das et al*.* (44), cytoplasmic proteins, but not organellar or membrane-associated ones, leak out from the parasites upon treatments with inhibitors against PfATP4 (a sodium pump) and saponin. Once PfATP4 is inhibited, saponin becomes effective on the parasite plasma membrane because of cholesterol accumulation (44). After the combined treatment of PfATP4 inhibitors and saponin, cytoplasmic proteins such as aldolase are no longer detectable in the parasite pellet. We reasoned that this cytoplasmic protein leakage assay is an ideal complementary approach to our fixed and live-cell microscopic data to verify whether PfsPPases are cytosolic, organellar, or both.

We first verified the localization of PfsPPases to the parasite’s mitochondrion utilizing the NF54attB-PfsPPases-mRuby^APT^ line. Briefly, the parasites were stained with green MitoTracker, treated with KAE609 (a PfATP4 inhibitor [45, 46]) for 2 h, and then subjected to saponin treatment. The treated parasites were immediately added to a poly-lysine-coated glass dish, incubated in culture media, and imaged under a fluorescence microscope for a short time (10–15 min). Meanwhile, MitoTracker-stained parasites without the KAE609/saponin treatment were imaged as controls. Live-cell microscopy revealed a strong fluorescence of PfsPPases in both the mitochondrion and cytoplasm of untreated parasites. Following the KAE609/saponin treatment, however, a strong fluorescence of PfsPPases was still detected in the mitochondrion but not in the cytoplasm anymore (Fig. 8B; additional images in Fig. S9A and B). This result confirms that PfsPPase (likely PfsPPase2) is localized to the parasite’s mitochondrion. On the other hand, to verify the localization of PfsPPases to the parasite’s apicoplast, we utilized the PfMev-PfsPPases-mRuby^APT^ parasite line and repeated the cytosolic protein leakage assay as described above. Live-cell microscopy also revealed a strong fluorescence of PfsPPases in both the apicoplast and cytoplasm of untreated parasites. Conversely, following the KAE609/saponin treatment, a strong fluorescence of PfsPPases was detected in the apicoplast but not in the cytoplasm anymore (Fig. 8C; additional images in Fig. S9C and D). This result confirms that PfsPPase (likely PfsPPase2) is also localized to the parasite’s apicoplast.

**Fig 9:**
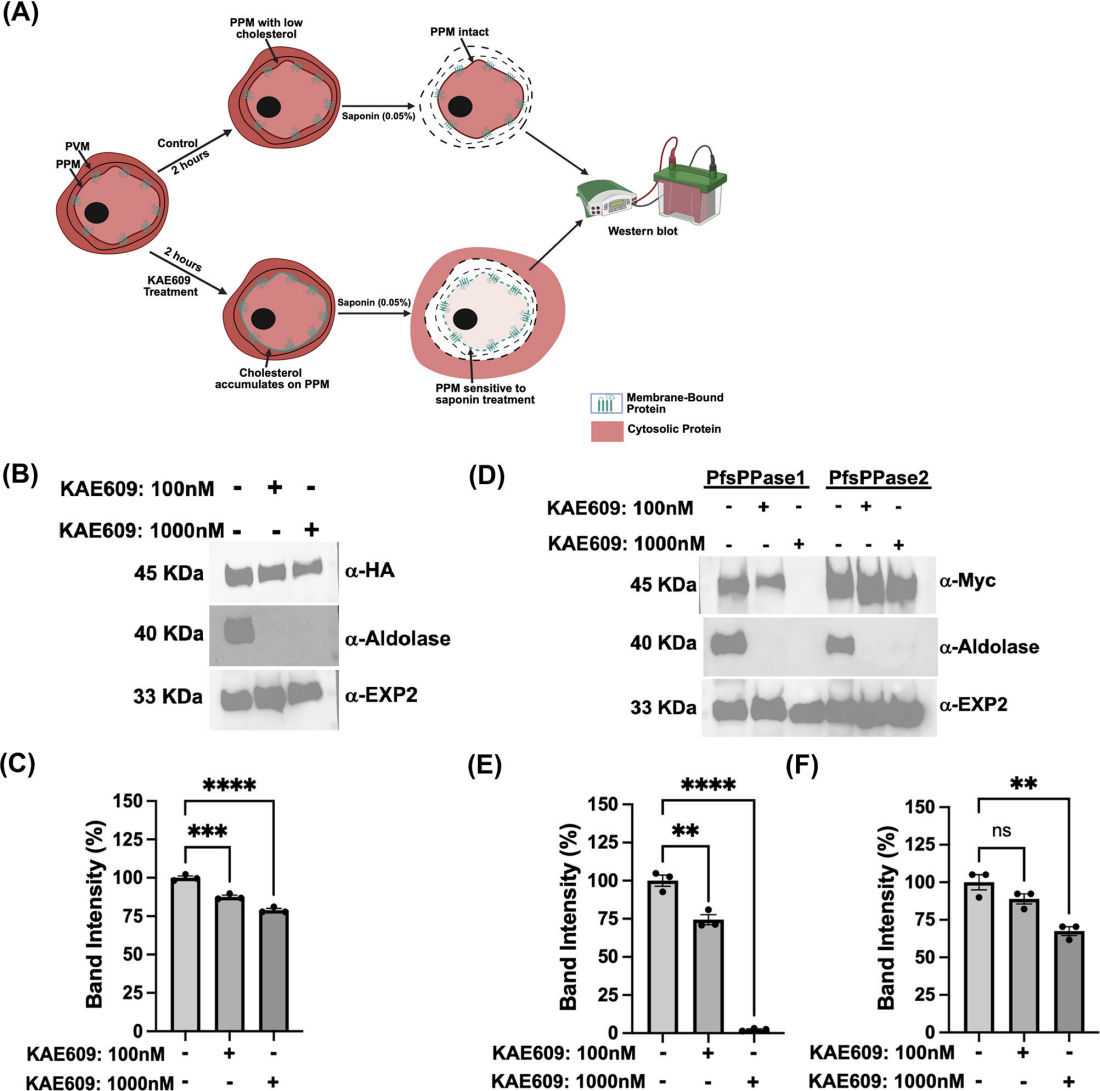
A cytoplasmic protein leakage assay shows PfsPPase1 is solely cytosolic, while PfsPPase2 localizes to organelles and cytoplasm. (**A**) Schematic diagram illustrating the cytosolic protein leakage assay for Western blot. The parasite lines of NF54attB-sPPase-3HA^APT^, PfsPPase1-3Myc, and PfsPPase2-3Myc were treated with KAE609 for 2 h, followed by saponin lysis and Western blot. (**B**) Western blot analysis of NF54attB-sPPase-3HA^APT^ treated with 0, 100, or 1,000 nM KAE609 for 2 h. The samples were probed with anti-HA, anti-PfAldolase, or anti-PfEXP2. (**C**) Band intensity measurement of B and two additional replicates with Image J. (**D**) Western blot analysis of PfsPPase1-3Myc and PfsPPase2-3Myc parasites treated with 0, 100, or 1,000 nM KAE609 for 2 h. (**E**) Band intensity measurement of PfsPPase1-3Myc using Image J. (**F**) Band intensity measurement of PfsPPase2-3Myc using Image J. Statistical analysis was done by *t*-tests. **, *P* < 0.05; ***, *P <* 0.001; ****, *P <* 0.0001; and ns, non-significant.

Furthermore, we repeated the cytosolic protein leakage assay using HA- and Myc-tagged parasite lines to trace PfsPPase proteins by Western blot (Fig. 9A). Briefly, NF54attB-PfsPPases-3HA^APT^ was treated with KAE609 at low and high concentrations for 2 h, saponin lysed, and subjected to Western blot with anti-HA antibody. Two control proteins were used to validate the assay, including the cytosolic PfAldolase (which should leak out) and the membrane-bound PfExp2 (which should be retained). Western blot revealed that KAE609/saponin treatment renders a complete leakage of PfAldolase, but no loss of PfExp2, ensuring that the protein leakage assay worked properly (Fig. 9B and C; Fig. S10A through C). PfsPPases were observed to be partially lost in the parasite pellet in a KAE609 concentration-dependent manner, but some protein remained even at the highest KAE609 concentration, suggesting that PfsPPases are cytoplasmic as well as organellar. To further validate the distinct localization of each PfsPPase isoform, we repeated the experiment using PfsPPase1-3Myc and PfsPPase2-3Myc lines at 96 h post-aTc removal to deplete the 3HA-tagged endogenous PfsPPases. Post-KAE609 treatment (2 h) and saponin lysis, the pellets were analyzed by Western blot, which revealed that PfsPPase1 was partially lost at a low concentration of KAE609 but was totally leaked out at the highest concentration. This strongly indicated that PfsPPase1 is exclusively cytosolic (Fig. 9D and E; Fig. S10D through F). In contrast, PsPPase2 showed no significant loss at a low concentration of KAE609 treatment and only a minor loss at the highest concentration (Fig. 9D and F; Fig. S10D through F), indicating that PfsPPase2 localizes to organelles as well as cytoplasm. Collectively, in combination with our fixed- and live-cell microscopic data, this cytosolic protein leakage assay strongly suggests that while PfsPPase1 is exclusively cytosolic, PfsPPase2 has cytosolic and organellar localization, further supporting the functional differences of the two PfsPPase isoforms.

One plausible explanation for PfsPPase2 being partially cytosolic is alternative translation initiation (ATI) of the PfsPPase2 transcript at amino acid 52 (methionine), which is the first amino acid of the PfsPPase1 transcript. ATI is a well-established mechanism by which multiple protein isoforms are generated from a single mRNA (47–49), facilitating protein trafficking to distinct subcellular compartments (49). To test the possibility that ATI at methionine 52 of PfsPPase2 generates PfsPPase1 proteins, we constructed a PfsPPase2M52A mutant (tagged by 3Myc) in the NF54attB-PfsPPases-3HA^APT^ parasite line, yielding a parasite line named NF54attB-PfsPPases-3HA^APT^-PfsPPase2M52A-3Myc (shortened as PfsPPase2M52A-3Myc). We reasoned that if ATI is critical, PfsPPase2M52A-3Myc would only generate organellar sPPases without any cytosolic counterparts; therefore, the mutant would fail to complement endogenous PfsPPases upon knockdown of both isoforms. We tightly synchronized PfsPPase2M52A-3Myc parasites, removed aTc, and verified that while endogenous PfsPPases-3HA were depleted upon aTc removal of 48 or 96 h, PfsPPase2M52A-3Myc was constitutively expressed (Fig. S11A and B). Under these conditions, parasites relied exclusively on PfsPPase2M52A-3Myc for PPi hydrolysis. Out of our expectation, however, PfsPPase2M52A fully sustained parasite growth and development, with no observable defects in parasite morphology or replication over four IDCs (Fig. S11C and D). Thus, the rescuing ability of PfsPPase2M52A is the same as that of wild-type PfsPPase2. We next examined the subcellular localization of PfsPPase2M52A by IFA, which revealed that in addition to the punctate organellar staining, diffuse cytosolic signal was also observed (Fig. S11E and F). This localization pattern again is the same as that of PfsPPase2. Altogether, these findings indicate that the cytosolic localization of PfsPPase2 is not dependent on ATI at methionine 52. It is plausible that PfsPPase2 gives rise to cytosolic sPPases through proteolytic cleavage at sites within its 51 aa leader sequence. At present, however, the exact cleavage sites and mechanisms of cleavage remain unknown.

## DISCUSSION

**Fig 10:**
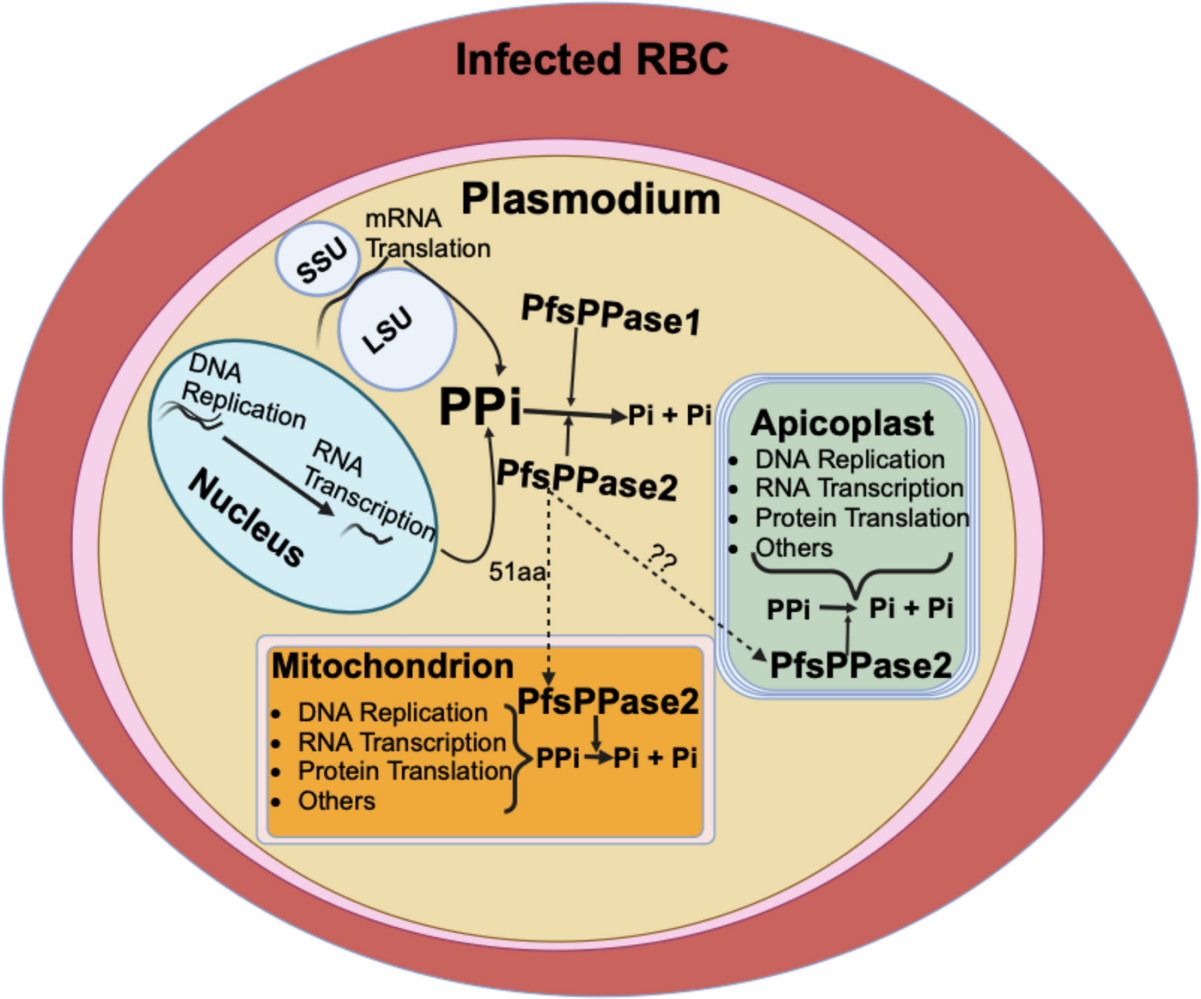
Schematic representation depicting the localization and functions of two PfsPPase isoforms in *Plasmodium falciparum*. PfsPPase1 is localized solely to the parasite cytoplasm, while PfsPPase2 displays a complex localization pattern, being present in the organelles (the mitochondrion and the apicoplast) and the cytoplasm. Two isoforms of PfsPPases work together to maintain pyrophosphate homeostasis in multiple subcellular compartments of *P. falciparum*. Question marks represent unknown mechanisms.

In this study, we employed reverse genetics, biochemical, and microscopic approaches to characterize soluble pyrophosphatases in *Plasmodium falciparum*, elucidating their roles during asexual blood stages. Our data indicate that *Plasmodium falciparum* encodes two sPPases from the same gene locus, and both PfsPPase1 and PfsPPase2 are essential for maintaining pyrophosphate metabolism in the cytoplasm and the organelles (mitochondrion and apicoplast) (see a model in Fig. 10). Despite being expressed throughout the asexual blood stages (18), knockdown of PfsPPases results in parasite arrest at the trophozoite stage, preventing its progression to the schizont stage (Fig. 2). We have previously demonstrated that the proton-pumping vacuolar pyrophosphatase (PfVP1) is essential in the ring stage (10). Together, our findings highlight that PPi homeostasis is essential in all asexual blood stages, with PPi hydrolysis mediated by distinct mechanisms—either via membrane-bound or soluble pyrophosphatases—at various stages of the 48 h IDC. Interestingly, despite being expressed in the late stages, PfVP1 is unable to compensate for the loss of PfsPPases. This suggests that the increased metabolic activities of the trophozoite stage (50–52) necessitate robust PPi degradation mediated by soluble pyrophosphatases.

Although the previous study characterized the enzymatic activities of PfsPPases in *P. falciparum* (18), our study is the first to report the essentiality of these enzymes and characterize the differences between the two isoforms. Both PfsPPase1 and PfsPPase2 are encoded by the same gene locus (Pf3D7_0316300) (Fig. 1) and play essential roles in PPi degradation during the asexual blood stages. The key difference between these isoforms is the presence of an additional 51-aa leader sequence in PfsPPase2 (Fig. 1A). Using multiple approaches, we have provided several lines of evidence supporting that both isoforms are independently transcribed and PfsPPase1 is not a product of alternative splicing from PfsPPase2. First, PCR amplifications using various primers and templates (DNA or cDNA) yielded specific PfsPPase1 amplicons that are independent of PfsPPase2 (Fig. 1). Second, Sanger sequencing confirmed that PfsPPase1 and PfsPPase2 possess distinct 5′ UTR (Fig. S2). Third, the 1.4 kb intron upstream of PfsPPase1 is sufficient to drive gene expression (Fig. S3). Although the specific sequences that constitute promoter elements in *Plasmodium* spp*.* remain poorly defined, our findings suggest that this 1.4 kb sequence works as a promoter for PfsPPase1. Intriguingly, this sequence is also an intron of PfsPPase2. We speculate that this hybrid feature or dual functionality of the 1.4 kb sequence is likely the mechanism by which one genetic locus, Pf3D7_0316300, encodes two proteins. A similar case was reported earlier in the gene locus that encodes both the stromal processing peptidase (SPP, Pf3D7_1440200) and the delta-aminolevulinic acid dehydratase (ALAD, Pf3D7_1440300) (53). The two genes share the same 5′ UTR, the first two exons, and the first intron. Intriguingly, the second long intron of Pf3D7_1440200 also encodes the remaining four exons of Pf3D7_1440300. Thus, our data and the published work reflect the complexity of the parasite genetic architecture.

We studied the localization of two PfsPPase isoforms by employing genetic tagging and multiple approaches in six transgenic parasite lines with different epitope tags (3HA, mRuby, or mNeonGreen) on either the full-length protein or the 51-aa leader sequence. Our data revealed that PfsPPase1 is exclusively cytoplasmic (Fig. 6 to 9), which is unable to fully rescue the PfsPPases knockdown parasites, as abnormal morphologies were observed by Day 6 post-aTc removal (Fig. 3A through C). We propose that the inability of PfsPPase1 alone to fully compensate for the loss of PfsPPases is because it lacks the 51-aa leader sequence, remains cytoplasmic, and likely allows PPi accumulation to occur in the organelles. By contrast, PfsPPase2 exhibits multiple localizations in the cytosol, mitochondrion, and apicoplast. Thus, PfsPPase2 alone is fully capable of maintaining PPi homeostasis in the entire parasite. While our measurements of PPi levels at 96 or 192 h post-knockdown suggest that both 3Myc-tagged PfsPPase isoforms work properly in the parasites (Fig. 4; Fig. S6), at present, we are unable to show direct organellar accumulation of PPi in the complemented lines. This is due to the lack of established protocols to completely purify mitochondria and apicoplasts from *P. falciparum* at high purity. Clearly, future studies are needed to confirm the accumulation of PPi in these organelles in the absence of organellar PfsPPases.

The localization data are consistent with the presence of three distinct genomes in *P. falciparum* in the nucleus (23 Mb) (19, 20), the mitochondrion (6 kb) (21–23), and the apicoplast (35 kb) (24–27), requiring PPi to be degraded by various mechanisms. PPi generated in the nucleus from various reactions can be shuttled through the nuclear pore complexes into the cytoplasm, where it is degraded by cytosolic pyrophosphatases along with PPi generated through cytosolic reactions. Prior to our study, it remained unknown how the mitochondrion and apicoplast deal with PPi homeostasis in malaria parasites, as no PPi transporters are annotated in the parasite genome, which is consistent with the lack of PPi transporters in model organisms (7, 16). Our results suggest that PfsPPase2 localizes to the mitochondrion and apicoplast to degrade PPi to monophosphate, which can then be exported by phosphate transporters on the organelles. Indeed, phosphate transporters are present on the membranes of parasite mitochondrion (54, 55), apicoplast (56–58), and plasma membrane (59), suggesting that parasites have mechanisms to freely exchange phosphate across different compartments.

Our findings reveal that *P. falciparum* utilizes a leader sequence to distribute sPPase enzymes into two organelles, a mechanism that has not been previously reported (Fig. S7). *S. cerevisiae* and mammals have two separate sPPase genes to encode proteins to be localized in the cytoplasm and mitochondrion (15, 16, 60). The two sPPase enzymes only share moderate sequence identity (47% and 62% identify for yeast [60] and human [15] enzymes, respectively). *Arabidopsis thaliana* distributes five sPPase isoforms with 69% identity to cytoplasm and mitochondrion; it also encodes a plastid pyrophosphatase, which is highly divergent from cytosolic and mitochondrial isoforms (61, 62). In comparison to these model organisms, *P. falciparum* has seemingly taken a unique route to duplicate the enzymes by adding a leader sequence in front of the soluble pyrophosphatase for degrading locally generated PPi in the organelles. The mitochondrial localization of PfsPPase2 is consistent with bioinformatic predictions (a score of 0.8 by MitoProt). Future studies will focus on how PfsPPase2 localizes to the apicoplast. It is likely that the leading sequence is sufficient for apicoplast targeting. Alternatively, PfsPPase2 can be trafficked to the apicoplast via mitochondrion-apicoplast contact sites or by other mechanisms. A study in *Toxoplasma gondii*, another apicomplexan parasite related to *Plasmodium*, demonstrated that proteins with dual localization to the apicoplast and mitochondrion can be translocated via Golgi-dependent pathways (63). Whether or not a similar mechanism applies to PfsPPase2 trafficking deserves further investigation.

Additionally, our data via cytoplasmic protein leakage assays suggest that a small portion of PfsPPase2 is cytosolic, which leaks out upon the combined treatment of KAE609 and saponin (Fig. 9). We speculate that while most PfsPPase2 proteins are organellar, some proteins become cytosolic after a proteolytic cleavage of the N-terminal sequence. Although direct evidence for this hypothesis is not yet available, we show that cytosolic PfsPPase2 is not a product of alternate translation initiation of methionine 52 because the M52A mutant behaved the same as the wild-type PfsPPase2, working properly to serve PPi homeostasis in three compartments (the cytosol, mitochondrion, and apicoplast) (Fig. S11). Furthermore, it should be noted that although two sPPase transcripts were confirmed by various means, we could only detect one protein band at the molecular weight of PfsPPase1, suggesting that a rapid cleavage of the 51-aa leader sequence from PfsPPase2 likely occurs during protein translocation, although the exact cleavage site and mechanism remain unknown.

Last but not least, we adopted the cytoplasmic protein leakage assay initially developed by the Vaidya group (44) and combined it with live-cell microscopy to differentiate whether PfsPPases are cytosolic or organellar in unfixed samples in real time (Fig. 8). This modified live-cell microscopy is widely applicable to study protein localization in *P. falciparum* if fluorescent tags are added to them, especially for proteins that display complex or multiple localizations. Leakage of the cytosolic portion upon the combined treatment of PfATP4 inhibitors and saponin will remove the cytosolic background, rendering the organellar signals better to be observed. Additionally, this novel method can also be used to study protein trafficking, especially for those that translocate through the cytosol. The development of this modified live-cell microscopy for protein localization is another significant contribution of this study.

In summary, our findings underline the critical role of PfsPPases in maintaining pyrophosphate homeostasis in the parasite’s cytosol and organelles (mitochondrion and apicoplast), a process crucial for maintaining intracellular balance and supporting parasite growth and development during the asexual blood stages, particularly at the trophozoite-to-schizont transition. The essentiality of PfsPPases, coupled with their divergence from human orthologs, underscores their potential to be a promising antimalarial drug target.

## MATERIALS AND METHODS

### Plasmid construction

Details of plasmid construction and confirmation can be found in Supplemental Information.

All primers used in this study were ordered from Azenta and listed in Table S1. Whole plasmid sequencing and Sanger sequencing were carried out by Azenta.

### Parasite culture, transfection, and knockdown studies

*P. falciparum* parasites were cultured in human O^+^ RBCs using RPMI-1640 media supplemented with 0.3% Albumax I as previously described (37, 64). Ring-stage parasites (~5% parasitemia) were transfected with either linearized or circular plasmids (50 µg) using a Bio-Rad electroporator. Forty-eight hours post-transfection, parasite cultures were maintained in media containing the appropriate drugs for selection, including blasticidin (2.5 µg/mL, InvivoGen) or WR99210 (5 nM, a kind gift from Jacobs Pharmaceutical). For parasites that need anhydrotetracycline (250 nM, Fisher Scientific), aTc was added on the day of transfection. For knockdown studies, parasites were synchronized via several rounds of alanine/HEPES (0.5 M/10 mM), isolated by Percoll at late stages, washed three times with 1× PBS to remove residual aTc (and Percoll), diluted with fresh RBCs, and cultured in media with or without aTc for several IDCs. The parasite lines generated in this study are listed in Table S2.

### Western blot

Infected RBCs were treated with 0.05% saponin/PBS supplemented with 1× protease inhibitor cocktail, and protein extraction was performed using 2% SDS/62 mM Tris-HCl (pH 6.8) as described previously (64). Following high-speed centrifugation (13,000 rpm, 10 min), the supernatant was used for protein electrophoresis. After electrophoresis, the proteins were transferred onto the blot, blocked with 5% non-fat milk in PBS, and incubated with primary antibodies as follows: anti-HA (1:10,000, mouse, sc-7392, Santa Cruz Biotechnology), anti-Myc (1:8,000, rabbit, 2278S, Cell Signaling), and anti-*Pf*EXP2 (1:10,000, rabbit, a gift from Dr. James Burns, Drexel University). The secondary antibodies used were HRP-conjugated goat anti-mouse (A16078, ThermoFisher Scientific) at 1:10,000 and HRP-conjugated goat anti-rabbit (31460, ThermoFisher Scientific) at 1:10,000. Subsequent steps followed the standard protocols. Blots were incubated with the Pierce ECL substrates and visualized using the ChemiDoc Imaging Systems (Bio-Rad). Protein concentration for all samples was determined using the detergent-tolerant Pierce BCA Protein Assay Kit (23227, ThermoFisher) according to the manufacturer’s instructions.

### Immunofluorescence assays

IFA was performed as described previously (37, 64). The following primary antibody was used: anti-HA (1:500, mouse sc-7392, Santa Cruz Biotechnology), anti-Myc (1:300, mouse 2276S, Cell Signaling), anti-PfACP (1:500, a gift from Dr. Sean Prigge, Johns Hopkins University), and anti-PfHSP60 (1:500, rabbit, NBP2-12734, Novus). Fluorescently labeled secondary antibodies for FITC or TRITC were used to incubate the samples for 3 h at room temperature. Other procedures followed the standard IFA protocol. Images were captured using the Nikon Ti microscope and processed by the NIS-Elements software.

### Live-cell fluorescence microscopy

An aliquot of infected RBCs (250 µL with 5% hematocrit and 5% parasitemia) was stained with Hoechst (1:1,000 dilution of 1 mg/mL), added into a glass-bottomed dish (P35G-1.5-14-C, Matteck), and incubated for 30 min. The dish was pre-treated with poly-lysine (P8920, Sigma) either at 4°C overnight or at 37°C for 1–2 h. After incubation in the dish, the extra non-adhered infected RBCs were washed off, and the adhered cells were added with 1 mL of phenol-free RPMI (R9002-01, US Biologicals) and then imaged under the Nikon Ti microscope.

### PPi extraction and measurement

PPi extraction was done in accordance with the published protocol with some modifications (65). Saline/glucose buffer (NaCl, 125 mM; KCl, 5 mM; MgCl_2_, 1 mM; glucose, 20 mM; and HEPES, 25 mM, pH 7.4*)* was used for saponin lysis and washing. In each condition, 2 × 10^8^ parasitized RBCs (or uninfected RBCs as a control) were saponin lysed and washed three times to remove hemoglobin, followed by the addition of 2 volumes of saline/glucose buffer. The mixture was heated at 90°C for 10 min to inactivate soluble pyrophosphatases and saved at −80°C. The samples were thawed and subjected to three freeze-thaw cycles between dry ice (10 min) and 37°C (~2 min), followed by sonication for 30 min at 4°C in a water bath sonicator (Fisher). After sonication, samples were spun down at 13,000 rpm for 10 min. The supernatants were saved for PPi measurement, while the pellets were solubilized with 2% SDS/62 mM Tris-HCl (pH 6.8) overnight for protein quantification. PPi was measured with a PPi fluorogenic sensor from Sigma (MAK168) in accordance with the manufacturer’s instructions. Briefly, 2 μL of each extracted supernatant was added to a 50 μL assay buffer containing the diluted PPi fluorogenic sensor (1:1,000) in a black plate. The mixture was incubated in the dark for 20–30 min and read by a plate reader (Tecan Infinite 200 Pro) based on the required excitation and emission wavelengths. A PPi standard curve was generated to determine PPi concentrations in samples.

### Cytoplasmic protein leakage assay for live-cell microscopy and Western blot

In this assay, NF54attB-PfsPPases-mRuby^APT^ and PfMEV-PfsPPases-mRuby^APT^ parasite lines were used for live-cell microscopy, while NF54attB-PfsPPases-3HA^APT^, PfsPPase1-3Myc, and PfsPPase2-3Myc lines were used for Western blot. In each condition, 1 × 10^7^ parasitized RBCs from each parasite line were treated with varied concentrations of KAE609 (45, 46) (100 and 1,000 nM for Western blot; 1,000 nM for live-cell microscopy) for 2 h. In the NF54attB-PfsPPases-mRuby^APT^ line, parasites were pre-stained with green Mitotracker (2263467, ThermoFisher) for 20 min before KAE609 treatment. Following KAE609 treatment, saponin was added directly to parasites (final concentration, 0.05%) and washed with 1× PBS twice. For microscopy, the saponin-treated parasites were transferred onto a poly-lysine-coated 35 mm glass bottom dish (MatTek, P35GC-1.5-14-C-HA) containing 1 mL of RPMI medium and imaged using the Nikon Ti fluorescent microscope for a short time (10–15 min). For Western blot, the saponin-treated parasite pellets were solubilized in 2% SDS/62 mM Tris-HCl (pH 6.8) and separated by SDS-PAGE. Anti-HA was used to detect PfsPPases-3HA, anti-MYC was used to detect PfsPPase1-Myc and PfsPase2-Myc, while anti-PfEXP2 (membrane-bound protein) and anti-PfAldolase (cytosolic protein) served as control proteins to validate the assay.

## Data Availability

The authors declare that all data of this work are included in the main article and Supplemental Information. Parasite lines and plasmids are available to the community upon request. Proper paperwork and MTA per institutional guidelines are needed prior to shipping the materials.

## SUPPLEMENTAL MATERIAL

Supplemental Information mbio.00475-26-s0001.doc Plasmid construction details, Tables S1 and S2, and Figures S1-S11.

ASM does not own the copyrights to Supplemental Material that may be linked to, or accessed through, an article. The authors have granted ASM a non-exclusive, world-wide license to publish the Supplemental Material files. Please contact the corresponding author directly for reuse.
