# Supplementary material for: Pyrophosphate homeostasis in multiple subcellular compartments is essential in Plasmodium falciparum
Source: mBio. 2026 Apr 22;17(5):e00475-26. doi: 10.1128/mbio.00475-26 (PMC13170353; doi:10.1128/mbio.00475-26)
Supplement: Supplemental Information — Plasmid construction details, Tables S1 and S2, and Figures S1-S11. [file mbio.00475-26-s0001.doc]

**Pyrophosphate homeostasis in multiple subcellular compartments**

**is essential in *Plasmodium falciparum***

Ikechukwu Nwankwo and Hangjun Ke

Authors Affiliation: Center for Molecular Parasitology, Department of Microbiology and Immunology, Drexel University College of Medicine, Philadelphia, Pennsylvania, United States of America

Keywords: *Plasmodium falciparum*, Malaria, pyrophosphate, soluble pyrophosphatase, cytoplasm, mitochondrion, apicoplast

Correspondence, Dr. Hangjun Ke, hk84@drexel.edu

**Running Title**

**Soluble Pyrophosphatases in *Plasmodium falciparum***

**Plasmid construction**

1, Cloning the 1.4 kb intronic region to drive yDHOD-GFP expression.

The 1.4-kb intronic region was amplified from parasite genomic DNA using the primers P6 and P7 and digested with ApaI and AvrII. The pLN-Bsd-CAM-yDHOD-GFP plasmid was similarly digested with the same enzymes to remove the calmodulin promoter. The digested insert and vector were ligated, yielding pLN-Bsd-1.4kb-yDHOD-GFP, which was confirmed by whole plasmid sequencing.

2, Endogenous tagging of PfsPPases with 3HA (hemagglutinin). To generate the transgenic parasite line NF54attB-PfsPPases-3HA^APT^, we modified the endogenous locus of PfsPPases (PF3D7_0316300) via CRISPR/Cas9 (1, 2). To construct the pMG75 vector plasmid, we amplified two homologous regions (5HR and 3HR) from *P. falciparum* genomic DNA using primers P8-P11. These two PCR products were annealed using an overlap extension PCR (3), forming a 3HR+5HR fused fragment. It was then digested with AflII and BstEII, cloned into the pMG75-3HA plasmid (4), and sequenced with the vector primers (P12-P13), yielding the pMG75-PfsPPases-3HA plasmid. For parasite transfection, we linearized the plasmid with SacII and mixed it with the circular gRNA plasmid. The gRNA sequence (P14) was selected using the Eukaryotic Pathogen CRISPR guide RNA design tool (http://grna.ctegd.uga.edu/) and cloned into our NFCas9 plasmid(4) via the NEB HiFi-DNA Assembly Master mix. The gRNA plasmid was confirmed by Sanger sequencing (P16).

3, Endogenous tagging of PfsPPases with mRuby. We used the previously PCR-amplified PfsPPases 3HR+5HR fragment, digested it with AflII and BstEII, and cloned it into the pMG75-PfVP1-mRUBY plasmid (a derivative of pMG75-PfVP1-mNeonGreen (5)). The correct insertion of 3HR+5HR was confirmed by sequencing (P19).

4, Generation of NF54attB-PfsPPases-3HA^APT^-PfsPPase1-3Myc and NF54attB-PfsPPases-3HA^APT^-PfsPPase2-3Myc parasite lines. In the NF54attB-PfsPPases-3HA^APT^ line, we performed a second transfection to complement the knockdown of PfsPPases with either PfsPPase1-3Myc or PfsPPase2-3Myc, driven by the mRL2 promoter (PF3D7_1132700) (6). Briefly, we amplified the full-length coding regions of PfsPPase1 or PfsPPase2 from parasite cDNA using primers P20 & P4 or P21 & P4, and individually cloned them into the pLN-mRL2-hDHFR-3Myc construct (7) via AvrII and BsiWI, yielding pLN-RL2-hDHFR-PfsPPase1-3Myc and pLN-RL2-hDHFR-PfsPPase2-3Myc, which were sequenced using the pLN vector primers (P22-P23).

5, Cloning the 51-aa leader sequence for localization studies. The fragment encoding the 51-aa leader sequence of PfsPPase2 was PCR amplified from parasite cDNA using primers P24 and P25. It was then cloned into the pLN-mRL2-Hsp60L-mNeongreen plasmid (8) by AvrII and NheI to replace the Hsp60 leader sequence. The correct insertion of the 51-aa leader sequence was confirmed by Sanger sequencing.

6. Cloning the PfsPPase2M52A mutant. Using the pLN-RL2-hDHFR-PfsPPase2-3Myc plasmid as template, we PCR amplified two fragments using P20&P25 (fragment 1) and P24&P4 (fragment 2). The two fragments were fused into one piece by overlapping PCR to encode the full length PfsPPase2M52A. It was digested with AvrII and BsiWI and ligated with the digested pLN vector. Fragment 1 contains the mutated sequence to facilitate M52A mutation (ATG-GCC) and a silent mutation (GGA-GGC, G53G). The mutated sequence GCCGGC is also a unique NaeI site which was used to screen bacterial colonies via plasmid isolation and restriction digestion as the new plasmid, but not the parental ones, was digestible by NaeI. The positive plasmid containing PfsPPase2M52A was further confirmed by whole plasmid sequencing.

**Supplementary Table 1: List of Primers**

| Primer | Name | Sequence |
| --- | --- | --- |
| P1 | IPP2-5UTR_294bp | CATGTTAACAGTGTAATCATATG |
| P2 | IPP1-5UTR_190bp | GAAGGCTAATTTGTGTGCGC |
| P3 | IPP1/2 Rev | AACGGCCGCCAGTGTGCTGGATGAACCCTAACCAAA |
| P4 | IPP1/2_BsiWI_Rev | ATCGTACGAGGTGTCCATATGTTGAGATCT |
| P5 | PfsPPases_Seq_Rev | GGATATTCATATGTTTGTGGTAAAG |
| P6 | PfsPPase1 Promoter-1.4 kb (Apal)-F | AGATCTTTTATTAACAAAAGTGTAAAA*GTATG*A |
| P7 | PfsPPase1 Promoter-1.4 kb (AvrII) -F | ATCCTAGGTATAATCTCCCAAATTTCTCTAAGA |
| P8 | PFSPPASE_KD_5HR_F | CCGCGGGATATCTATTCCGGAATATTGGGAGCTTTTACGTTGA |
| P9 | PFSPPASE_KD_5HR_R | CTGGTAACCTGTGGAGTCCAAATATTTAAGTCAGGTTTATAAGCACTATCAGACTTATAG |
| P10 | PFSPPASE_KD_3HR_F | CTCTTAAGGTATATATTTCATTTTGCTATACTATTG |
| P11 | PFSPPASE_KD_3HR_R | TCCGGAATAGATATCCCGCGGGAAACAAAGTATGAAAGGATATG |
| P12 | PMG75SEQ_F_2^ND^ | CTATATACTATGGCTAAATATATAC |
| P13 | PMG75BBHA_R | GTCAGGAACGTCGTATGGATA |
| P14 | PFSPPASE_KD_GRNA1 | TCATATTAAGTATATAATATTGGTGTCCATATGTTGAGATCGTTTCAGAGCTATGCTGGA |
| P16 | SuperlongCPF | CTTTTATTTTTACTGTAATATAATTTTTTATGTAAAAATAAGGGG |
| P17 | PFSPPASE_KD_5FCHECK | ACAAGTGGTTCCGGTCAAG |
| P18 | PFSPPASE_KD_3FCHECK | CTGAAAATAAATGTAGTAGAGGT |
| P19 | MRUBY REV | CGCATATTTTCCTTGATCAGCTCTTC |
| P20 | PfIPP1_AvrII_F | ATCCTAGGATGGGAAGTAAACTTATAAATGTTG |
| P21 | PfIPP2_AvrII_F | ATCCTAGGATGTATATATATATTTTTTTGTGTATATGC |
| P22 | PLN5PRSEQ_RL2 | GTAAGTTCATTTTACCAGTTAAG |
| P23 | PLN3PRSEQ | GTAGACCCCATTGCGAGTAC |
| P24 | PfsPPaseL51aaAvrII_F | atcctaggATGTATATATATATTTTTTTGTGTATATG |
| P25 | PfsPPaseL51aaNheI_R | atGCTAGCCTTATTTTTATTATAATCTCCCAAATTTC |
| P26 | PfsPPase2M52A -F | GTAAACTTATAAATGTTGAAGGTGGCAATAATCAGGATGA |
| P27 | PfsPPase2M52A -R | CACCTTCAACATTTATAAGTTTACTGCCGGCCTTATTTTTATTATAATC |

**Supplementary Table 2. List of parasite lines generated in this study.**

| # | **Parasite line** | **Goal** |
| --- | --- | --- |
| 1 | NF54attB-1.4kb-yDHOD-GFP | To test whether the 1.4 kb intron works as a promoter |
| 2 | NF54attB-PfsPPases-3HA^APT^ | To test the essentiality and localization of PfsPPases |
| 3 | NF54attB-PfsPPases-3HA^APT^-PfsPPase1-3Myc | To determine the function and localization of PfsPPase1 |
| 4 | NF54attB-PfsPPases-3HA^APT^-PfsPPase2-3Myc | To determine the function and localization of PfsPPase2 |
| 5 | NF54attB-PfsPPase2^leader^-mNeonGreen | To verify the organellar localization of PfsPPase2 |
| 6 | PfMev-PfsPPases-mRuby^APT^ | To confirm the PfsPPase localization to the apicoplast |
| 7 | NF54attB-PfsPPases-mRuby^APT^ | To confirm the PfsPPase localization to the mitochondrion |
| 8 | NF54attB-PfsPPases-3HA^APT^-PfsPPase2M52A-3Myc | To test if PfsPPase2 can generate PfsPPase1 via alternative translation initiation |


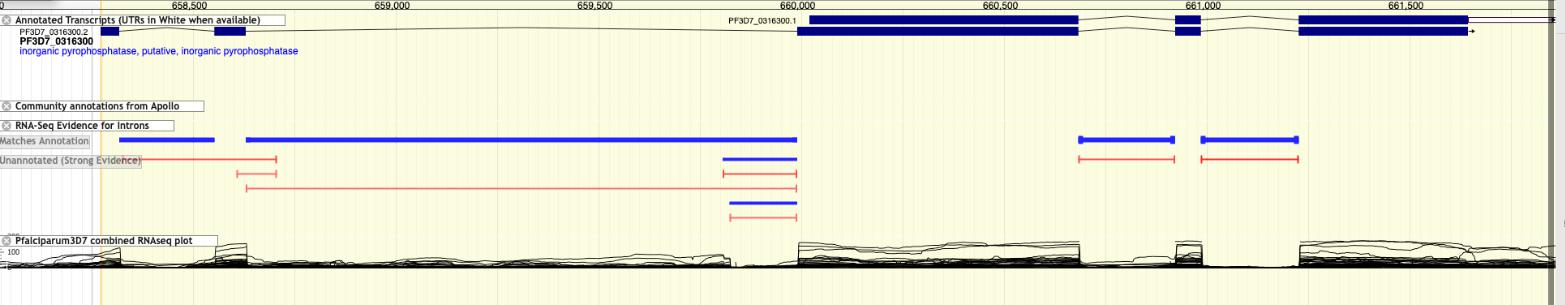


mmlsll,s,,

**Supplementary Figure 1. RNA-seq plot data for PfsPPases.**



RNA-seq plot showing the RNA-seq evidence for the introns and exons in the PfsPPase gene locus. Data was derived from PlasmoDB (www.PlasmoDB.org).

**(A)** **(B)**

**
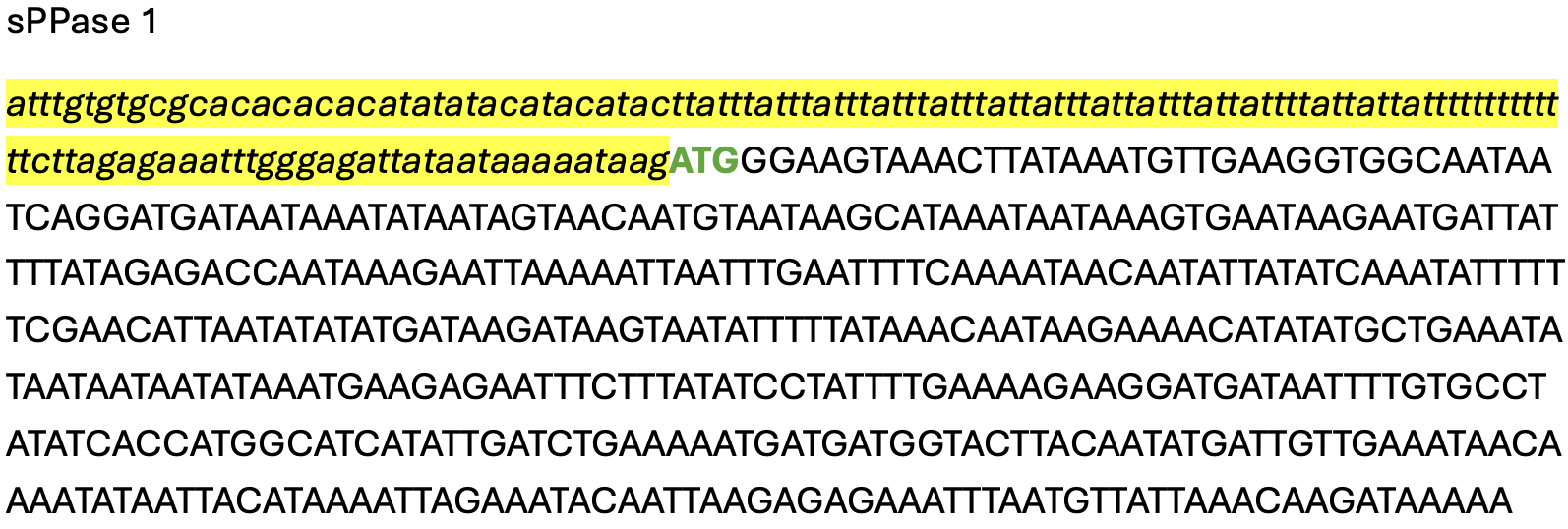

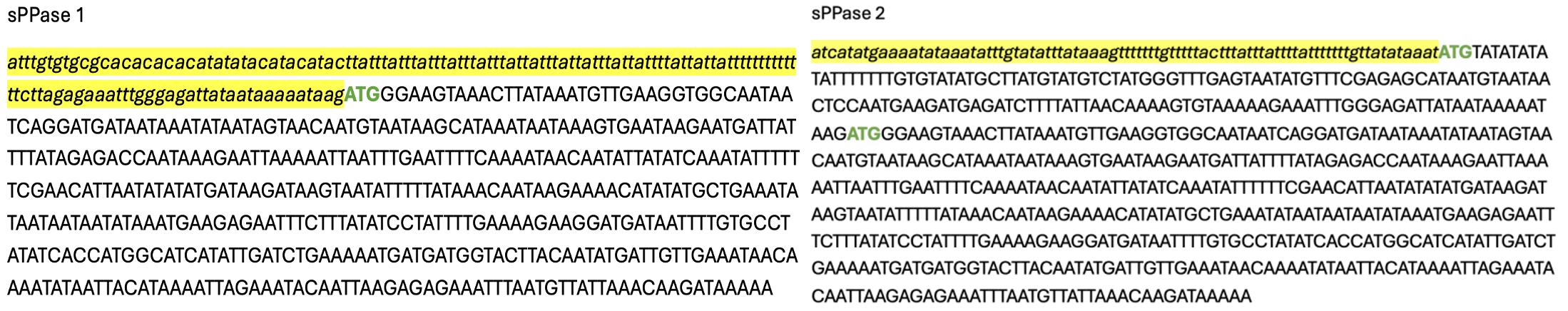

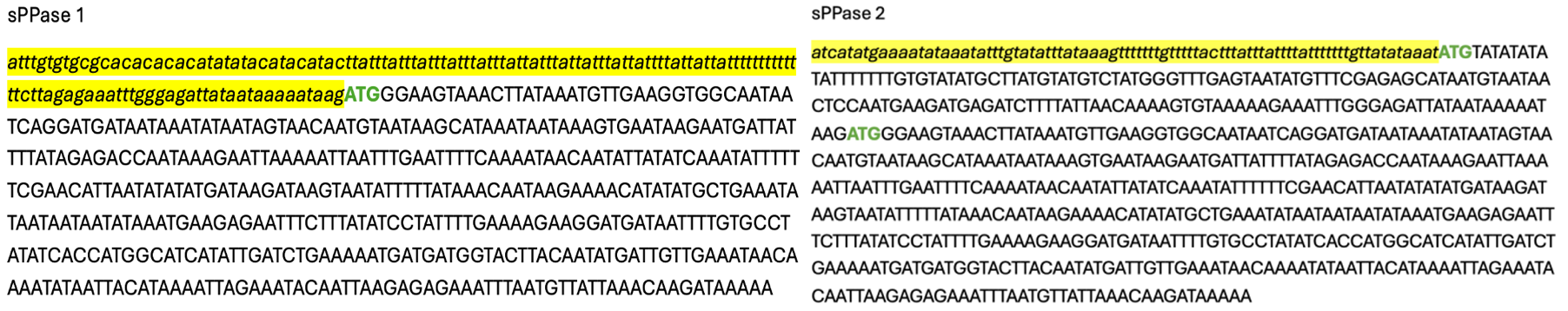
**

**(C)**

**
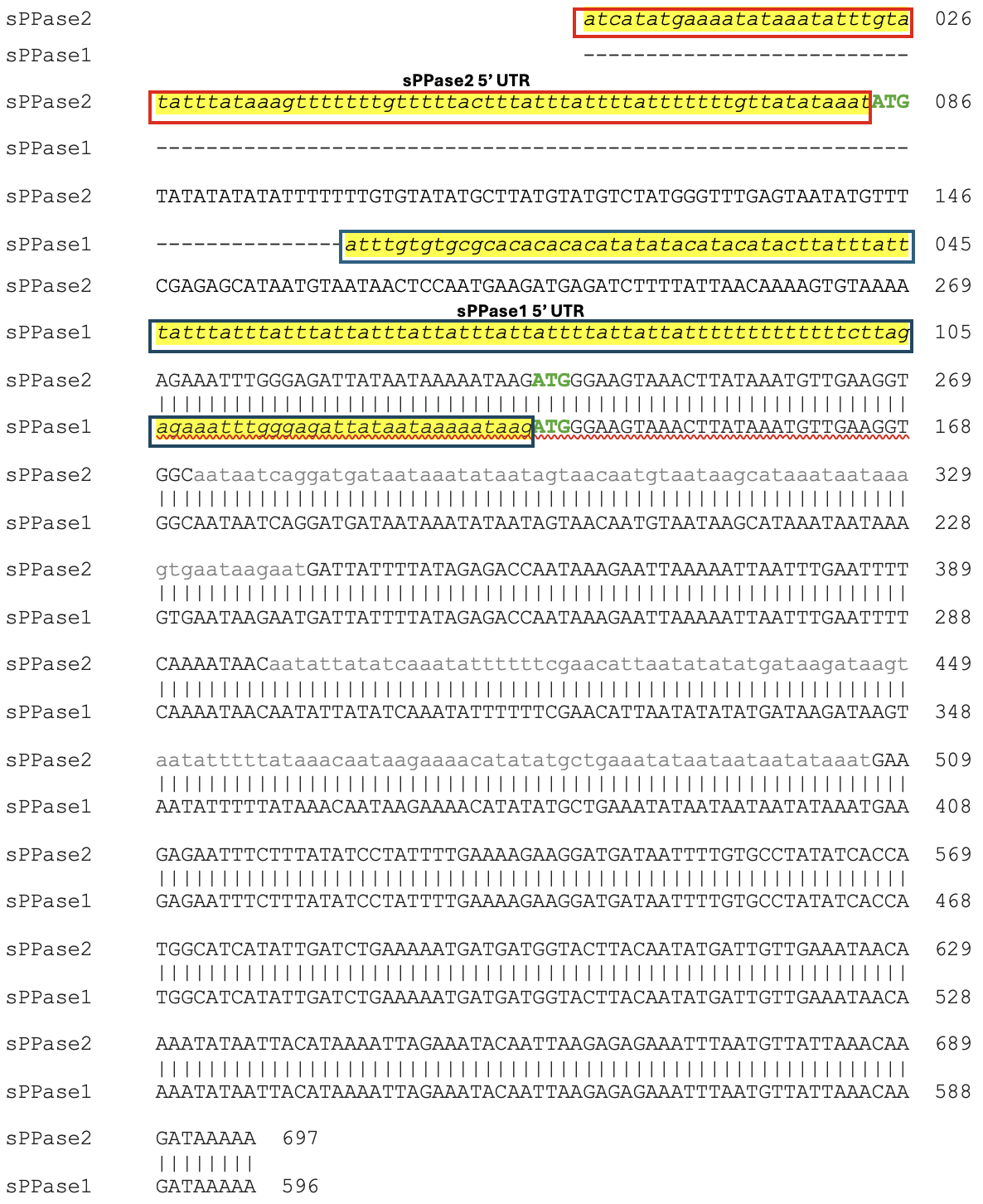
**

**Supplementary Figure 2. PfsPPase isoforms contain distinct 5’ untranslated regions.**

A-B, Sequencing results of the two PfsPPase transcripts demonstrate that each transcript possesses distinct 5’ UTR, consistent with independent transcriptional events. C, Alignment of the PfsPPase1 and PfsPPase2 sequencing results.


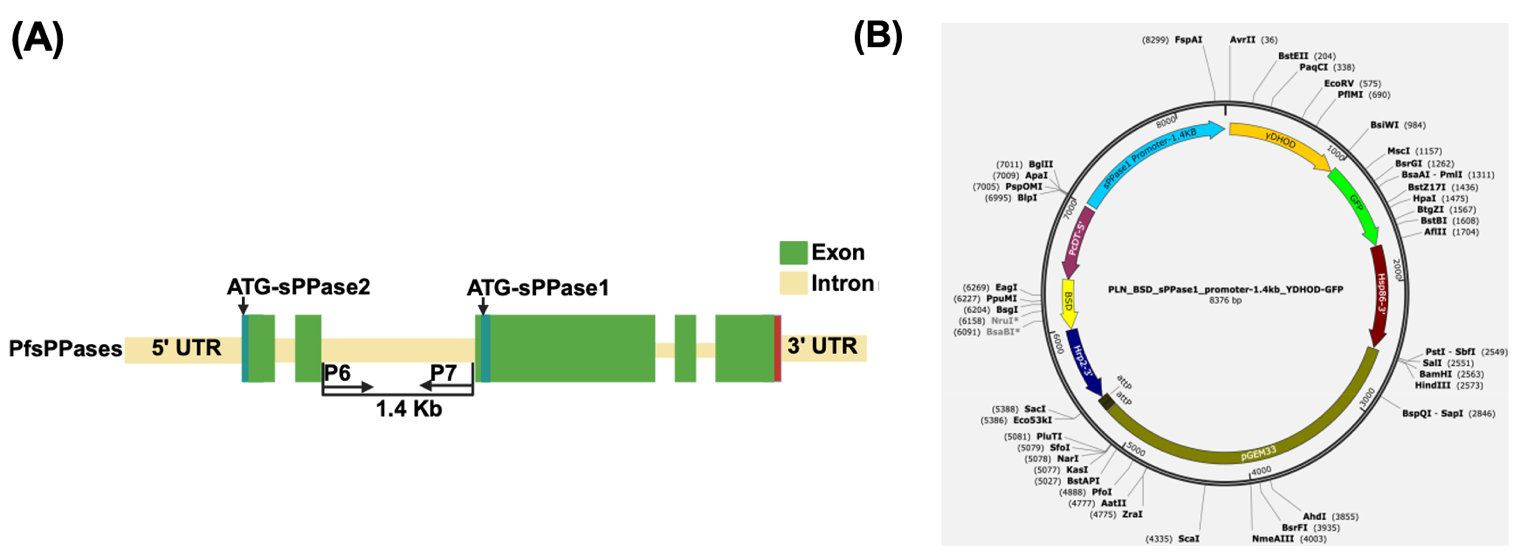


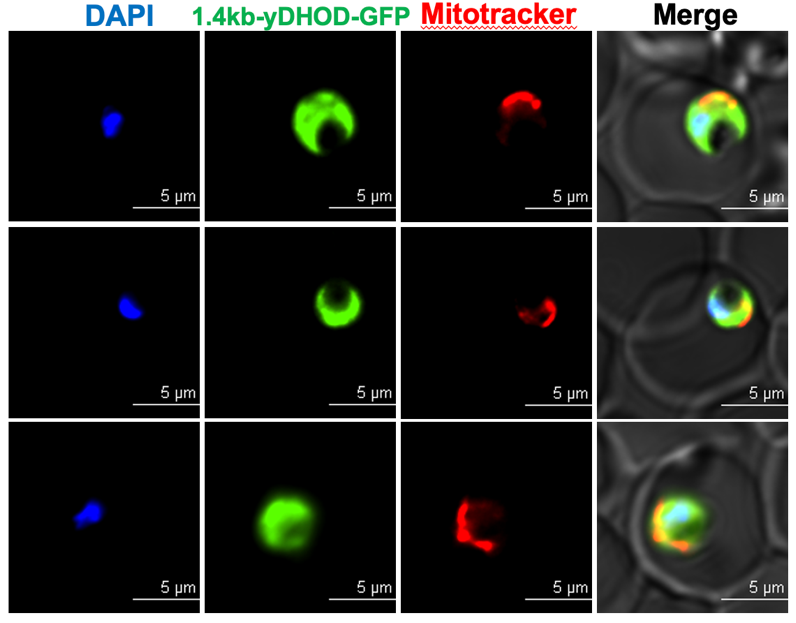
**(C)**

**Supplementary Figure 3. The 1.4 kb intron upstream of PfsPPase1 works as a promoter.**

A, Schematic diagram illustrating the position of the 1.4 kb sequence within the gene locus of PfsPPases. B. The plasmid map of pLN-BSD-1.4kb-yDHOD-GFP, showing the 1.4 kb sequence was properly cloned. C, Live imaging of NF54attB-1.4kb-yDHOD-GFP parasites, showing robust expression GFP. This indicates that the 1.4 kb sequence works as a promoter. DAPI stained the nuclei. Green, yDHOD-GFP. Mitotracker stained the mitochondrion. Scale bar 5 µm.


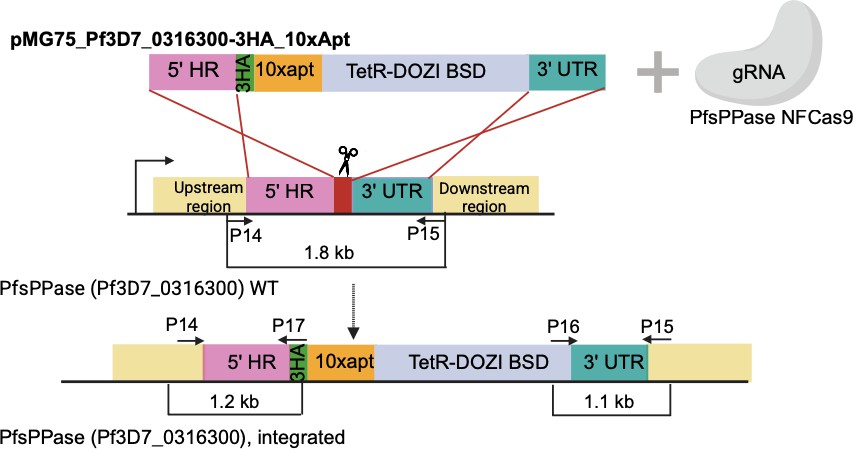
(A)


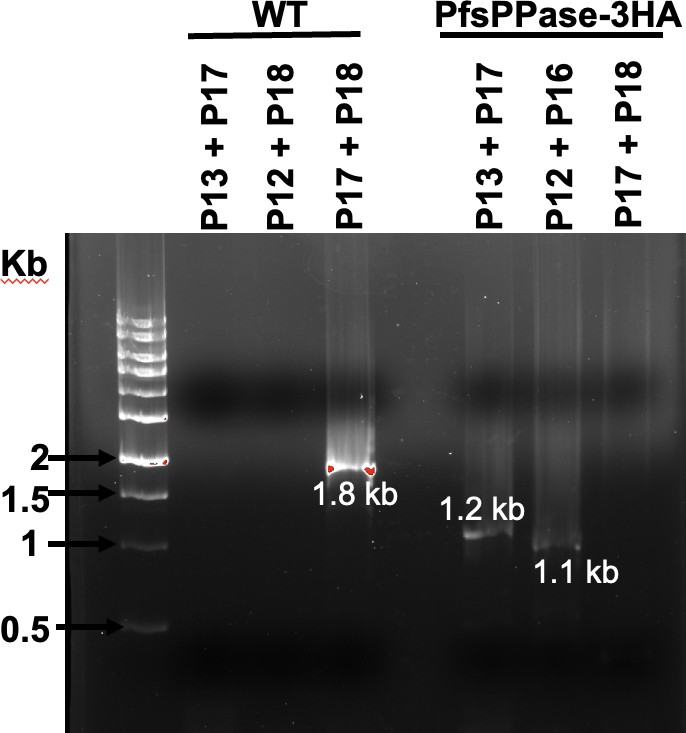
(B)


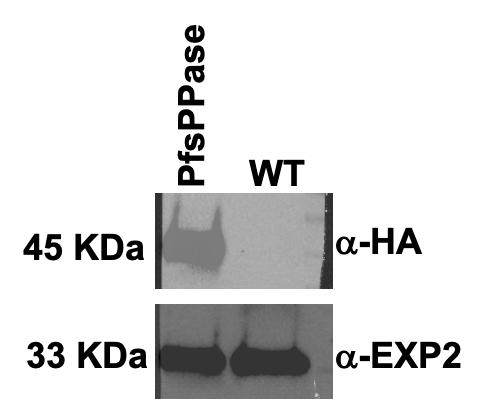

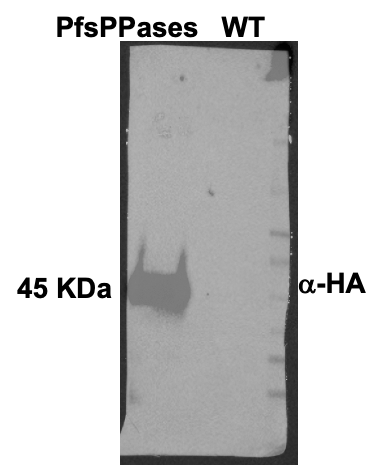

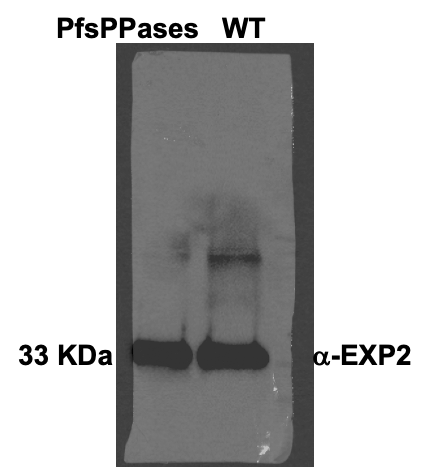


**(C)**

**(D)**

**(E)**

Supplementary Figure 4. CRISPR/Cas9 mediated genetic tagging of PfsPPases with 3HA and TetR-DOZI-aptamer system for conditional expression.

A, Schematic diagram illustrating endogenous tagging of the PfsPPases locus with 3HA (hemagglutinin) in the parasite line NF54attB. B, Diagnostic PCR shows proper integration of the pMG75_PfsPPases-3HA construct into the PfsPPases locus. C, Western blot confirming expression of PfsPPases-3HA at the expected size. D-E, Raw western blot images for panel C.

**(A)** **(B)**

**
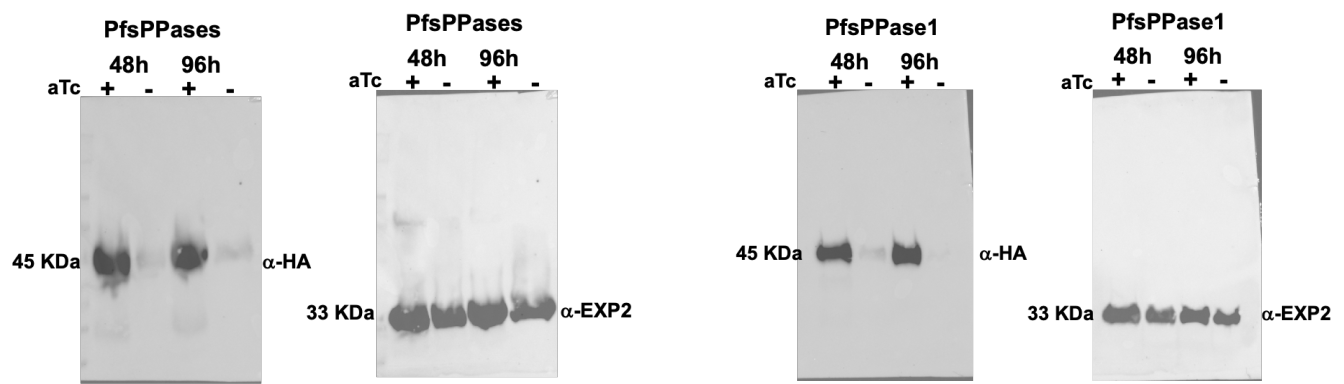
**

**(C)** **(D)**

**
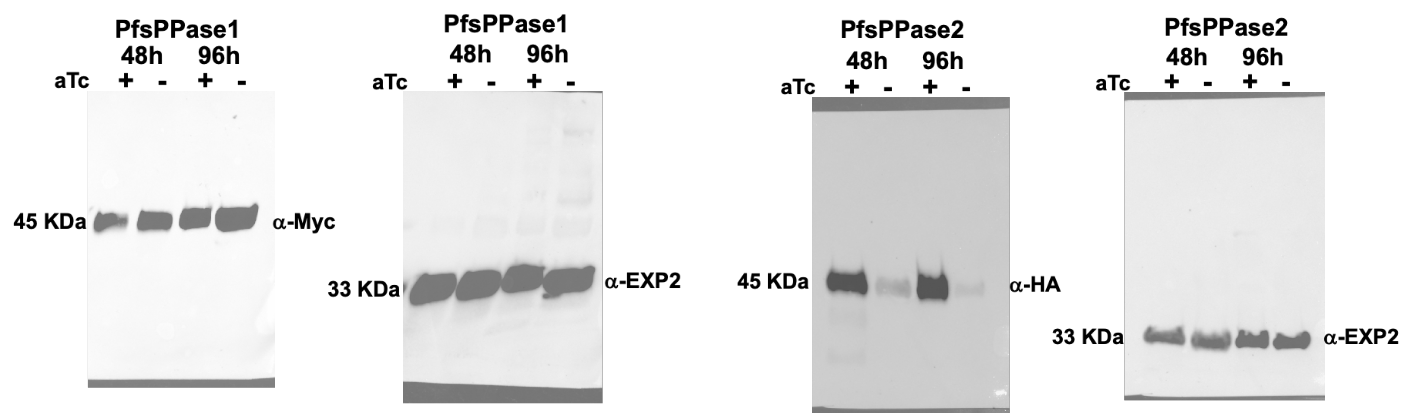
**

**(E)**

**
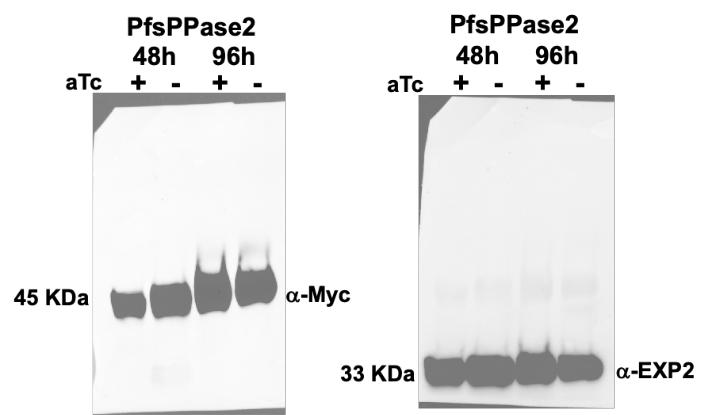
**

**Supplementary Figure 5. Raw Western blot.**

A, Raw western blot for Figure 2A. B-C, Raw western blot for Figure 3A. D-E, Raw western blot for Figure 3D.

1. **(B)**

**
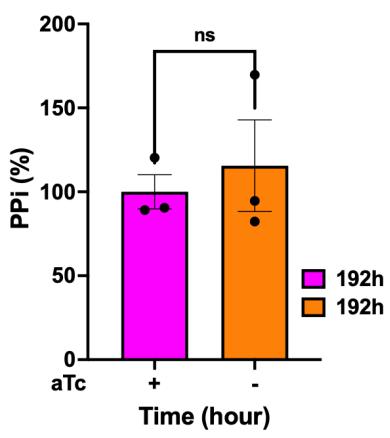

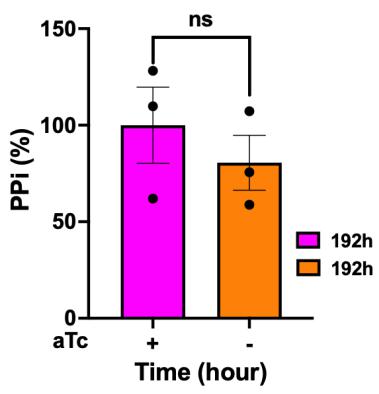
**

**Supplementary Figure 6. Quantifying PPi levels in PfsPPase1 and PfsPPase2 complemented lines.**

A-B, Measurement of PPi levels in the PfsPPase1-3myc and PfsPPase2-3myc respectively after aTc removal for 192h (4 IDCs) from the schizont stage. PPi concentration was measured and adjusted as nanomoles/mg and normalized to the control sample. Mean ± SEM of the 3 measurements is shown. Statistical analysis was done by unpaired t test. A, p value (0.1186); B, p value (0.1376).


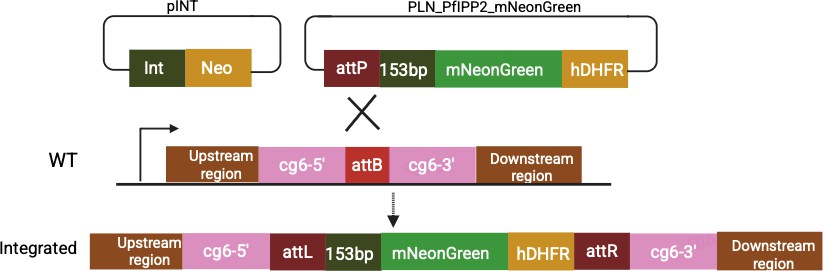
(A)

**(B)**

**
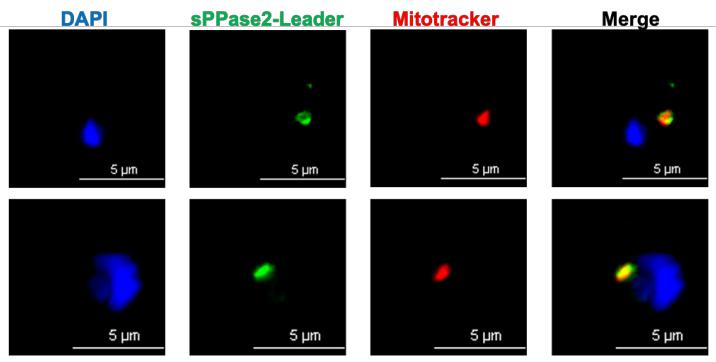
**




**(C)**

**
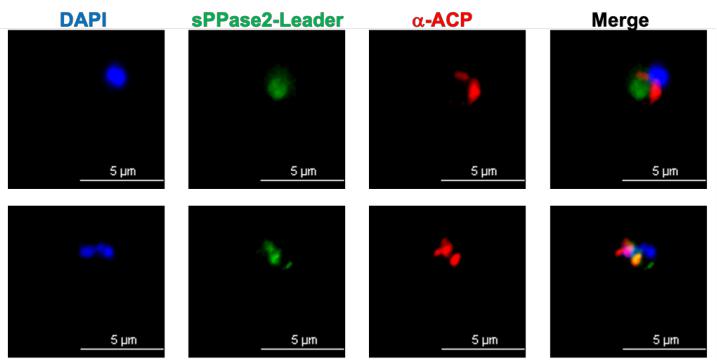
**

**Supplementary Figure 7. The 51-aa leader sequence of PfsPPase2 guides proteins into both the mitochondrion and the apicoplast.**

A, Schematic diagram illustrating the attBXattP integration system. The 51 amino acid (153bp) leader sequence at the N-terminal of PfsPPase2 was tagged with mNeonGreen and inserted into the genome of NF54attB parasites. B. Live imaging of NF54attB-PfsPPase2^leader^-mNG parasite stained with mitoTracker. DAPI stained the nuclei. Mitotracker stained the mitochondrion. Scale bar 5 µm. C, Immunofluorescence assay of NF54attB-PfsPPase2^leader^-MNG stained with anti-PfACP. DAPI stained the nuclei. Anti-PfACP stained the apicoplast. Scale bar 5 µm.

1.
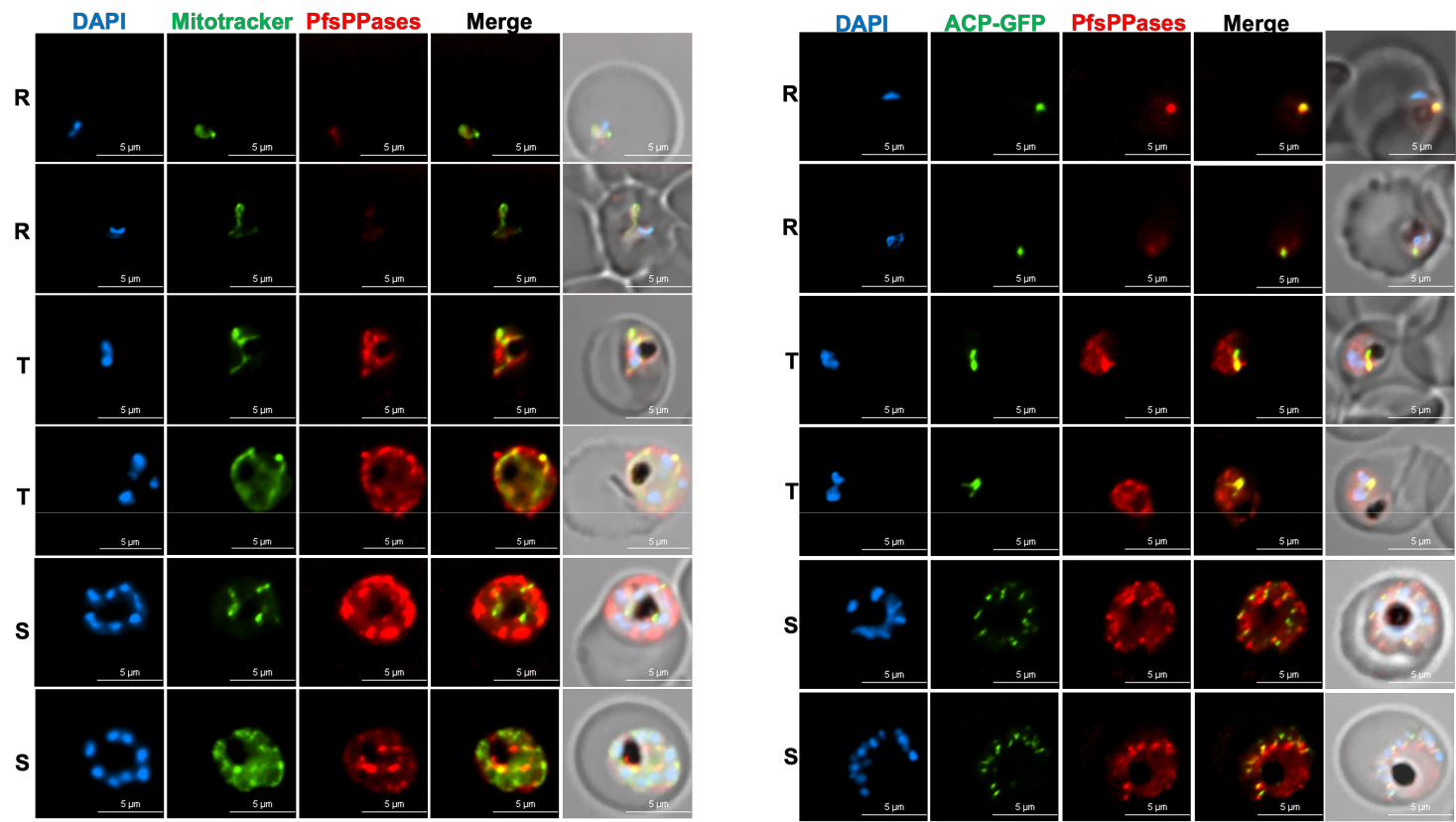
 **(B)**

**Supplementary Figure 8.** **Additional images showing the subcellular localization of PfsPPases via live microscopy**.

Live imaging of NF54attB-PfsPPases-mRuby (A) and PfMEV-PfsPPases-mRuby (B) showing expression and localization of the enzymes throughout the parasite’s IDC. R- Ring, T- Trophozoite, S- Schizont. DAPI stained the nuclei. Red, PfsPPases-mRuby. A, Colocalization of PfsPPases with the mitochondrion detected by green MitoTracker. Scale bars, 5 μm. Pearson correlation coefficient of green and red fluorescence was derived from n = 10 parasites of each stage. Ring (0.8967 ± 0.0489), Trophozoite (0.8252 ± 0.0432), and Schizont (0.8303 ± 0.0563). B, Colocalization of PfsPPases with the apicoplast labeled by GFP (guided by the first 55-aa of acyl carrier protein). Scale bars, 5 μm. Pearson correlation coefficient of green and red fluorescence was derived from n = 10 parasites of each stage. Ring (0.8609 ± 0.0664), Trophozoite (0.8599 ± 0.0833), and Schizont (0.8000 ± 0.0766).


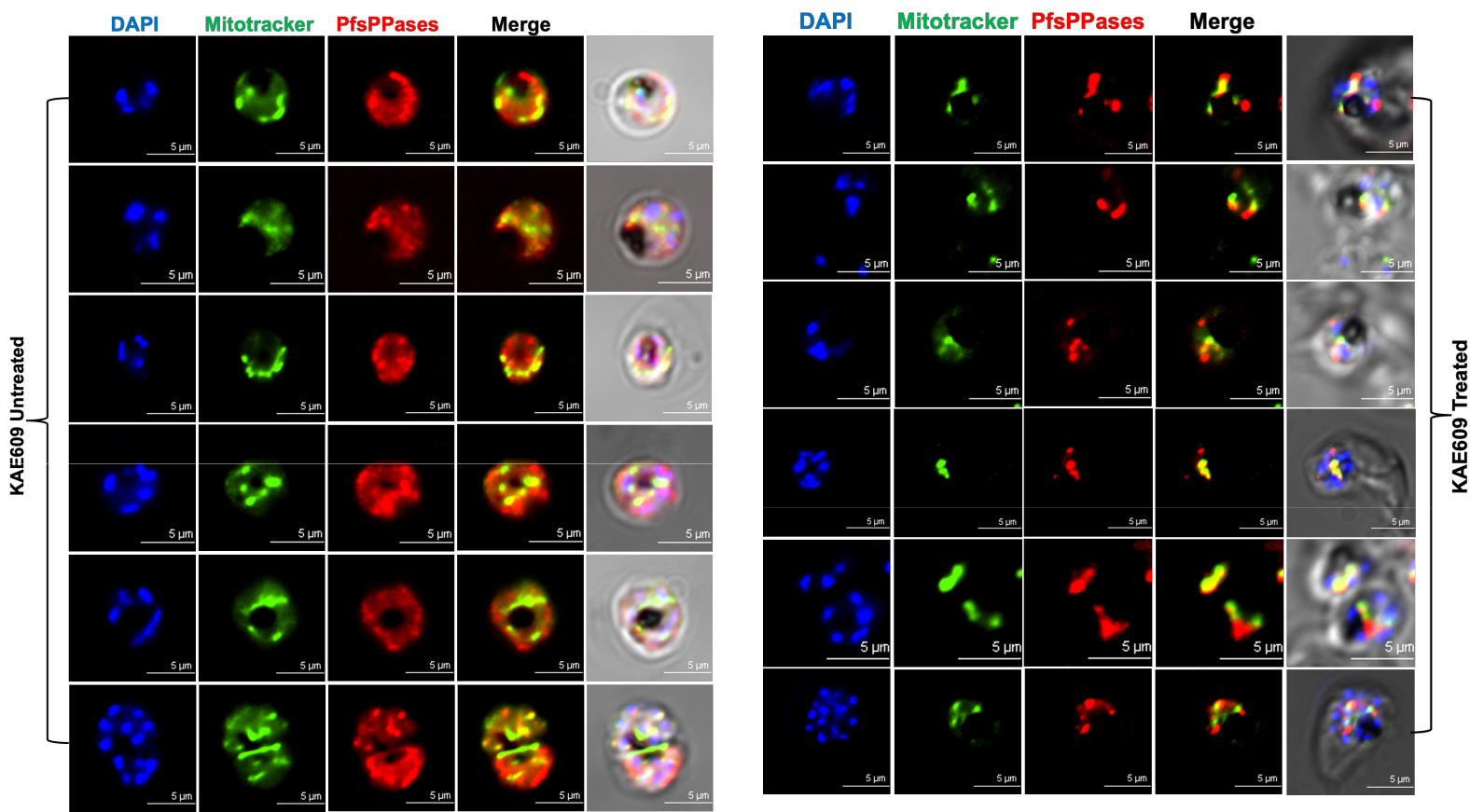
**(A)** **(B)**


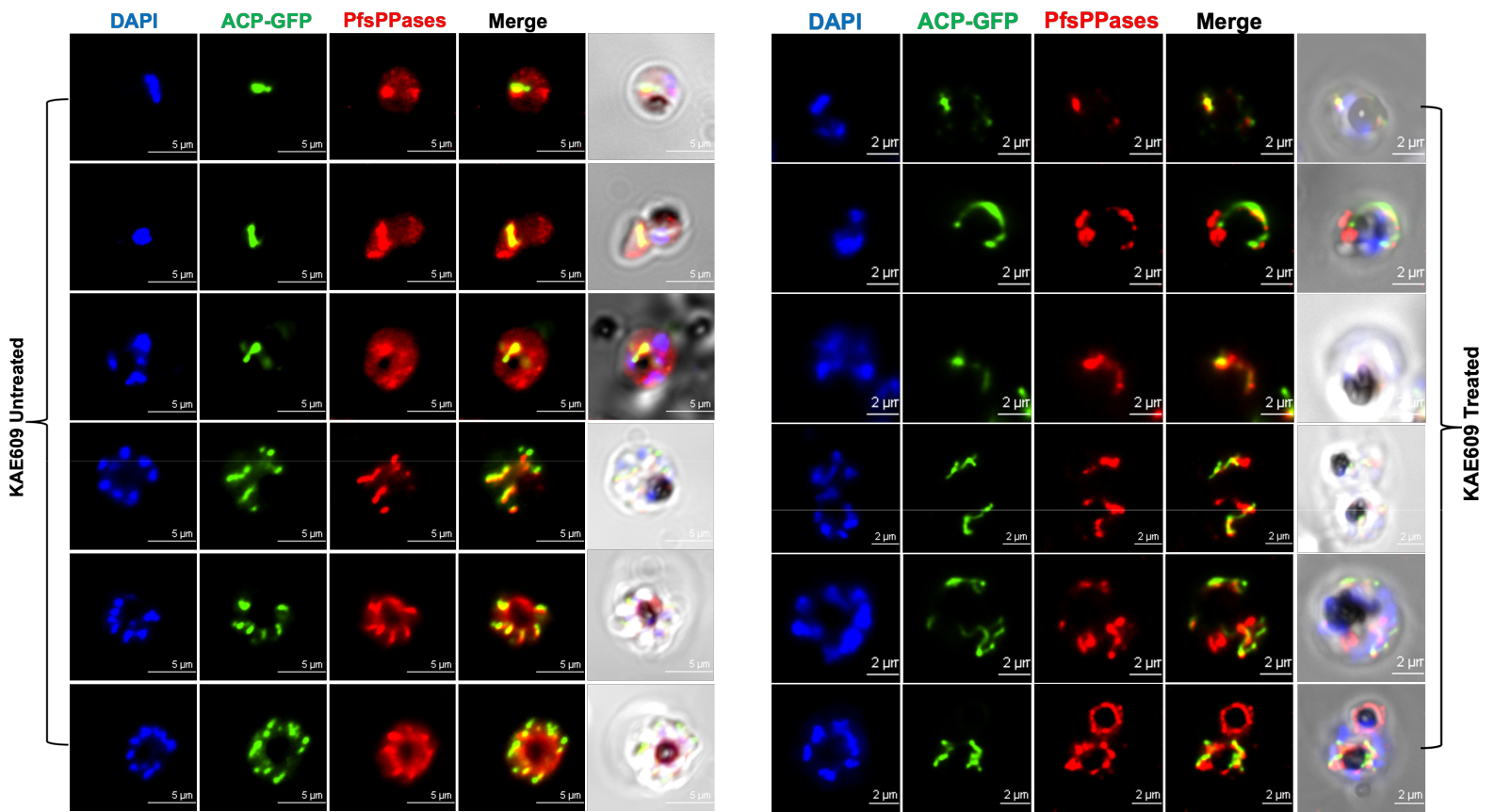
 **(C)** **(D)**

**Supplementary Figure 9. Additional images confirming the subcellular localization of PfsPPases from the cytoplasmic leakage assay.**

A-B, Colocalization of PfsPPases with the mitochondrion detected by green MitoTracker after cytosolic leakage assay using NF54attB-PfsPPases-mRuby parasites. A, KAE609 untreated. Scale bars, 5 μm. Pearson correlation coefficient of green and red fluorescence was derived from n = 10 parasites of each stage. Trophozoite (0.8241 ± 0.0680), and Schizont (0.8549 ± 0.0775). B, KAE609 treated. Scale bars, 5 μm. Pearson correlation coefficient of green and red fluorescence was derived from n = 10 parasites of each stage. Trophozoite (0.9095 ± 0.0980), and Schizont (0.8998 ± 0.0895). C-D, Colocalization of PfsPPases with the apicoplast labeled by GFP (guided by the first 55-aa of acyl carrier protein) after cytosolic protein leakage assay using PfMEV-PfsPPases-mRuby parasites. C, KAE609 untreated. Scale bars, 5 μm. Pearson correlation coefficient of green and red fluorescence was derived from n = 10 parasites of each stage. Trophozoite (0.8666 ± 0.0540), and Schizont (0.8087 ± 0.0877). D, KAE609 treated. Scale bars, 2 μm. Pearson correlation coefficient of green and red fluorescence was derived from n = 10 parasites of each stage. Trophozoite (0.8999 ± 0.0623), and Schizont (0.8021 ± 0.0566).

**(A)** **(B)** **(C)**

**
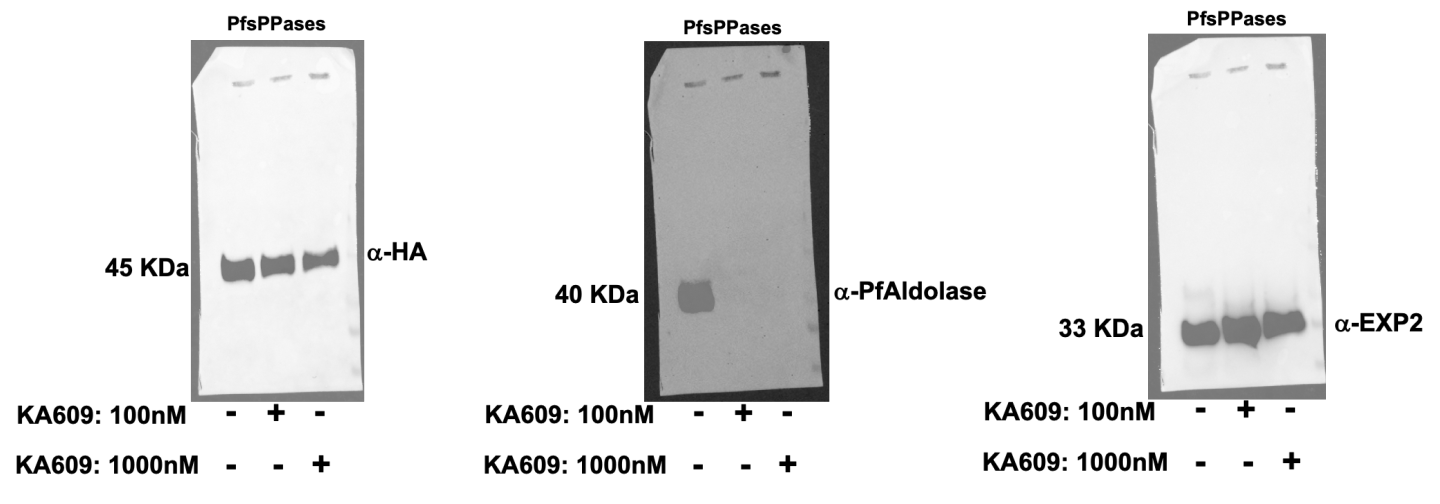
**

**(D)** **(E)**

**
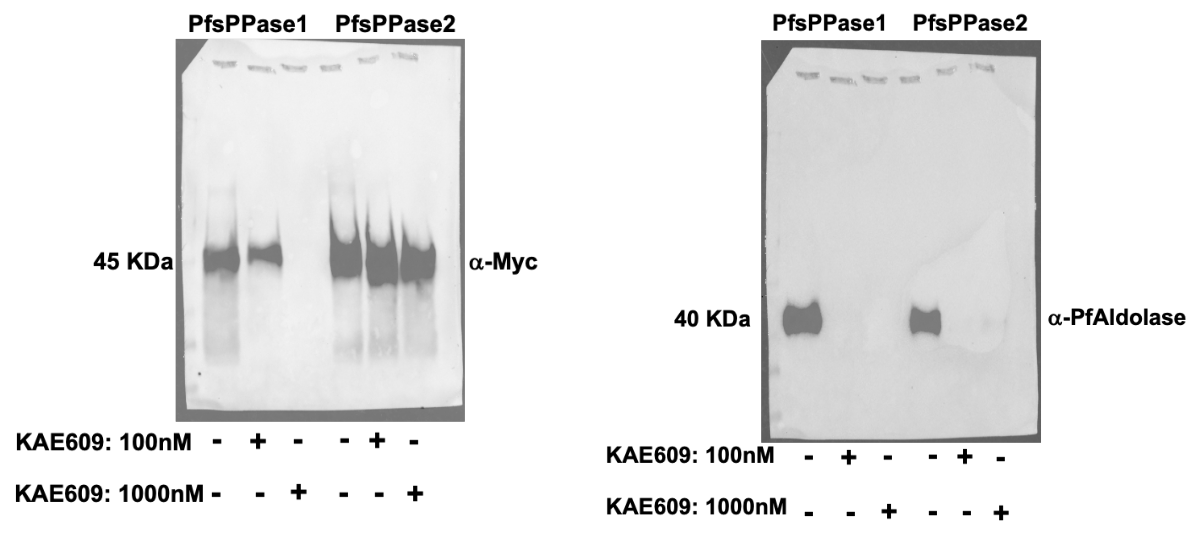
**

**(F)**

**
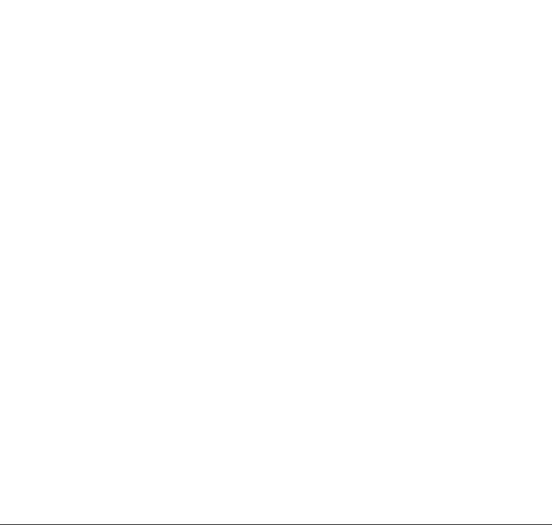

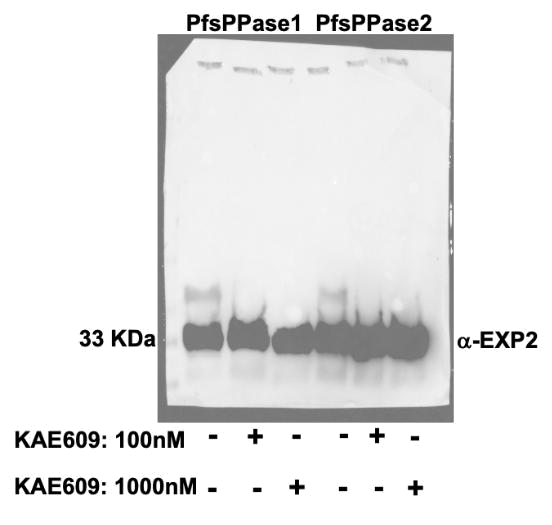
**

**Supplementary Figure 10. Raw Western blot.**

A-C, Raw western blot for Figure 9A. D-F, Raw western blot for Figure 9C.

**(A)** **(B)**


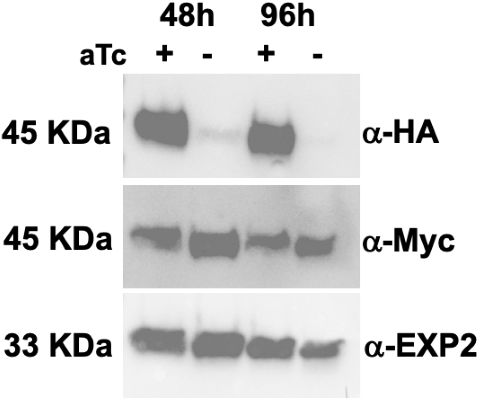

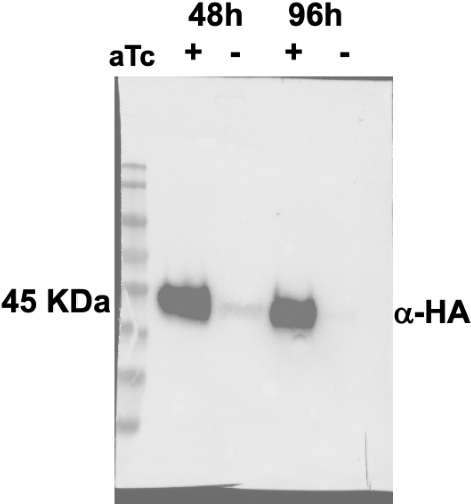

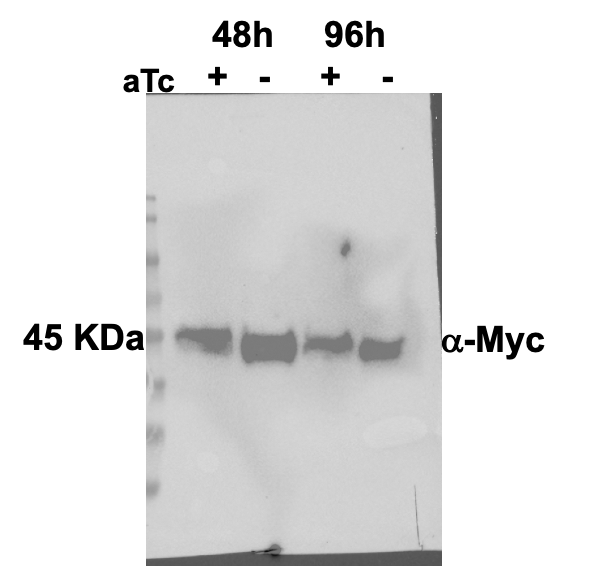

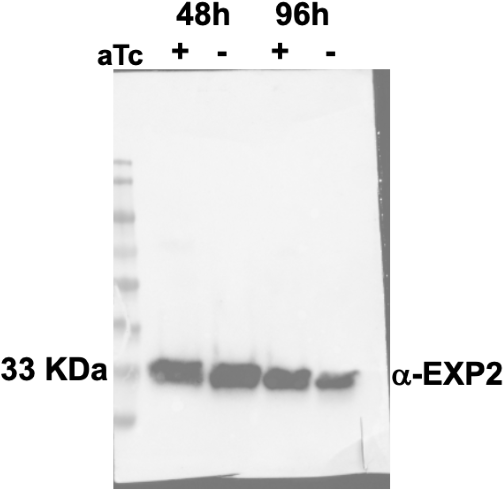


(

ii

)

(

iii

)

(

i

)


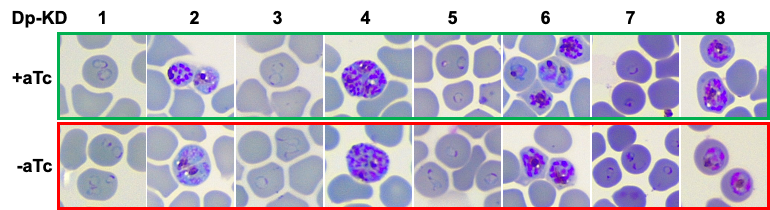

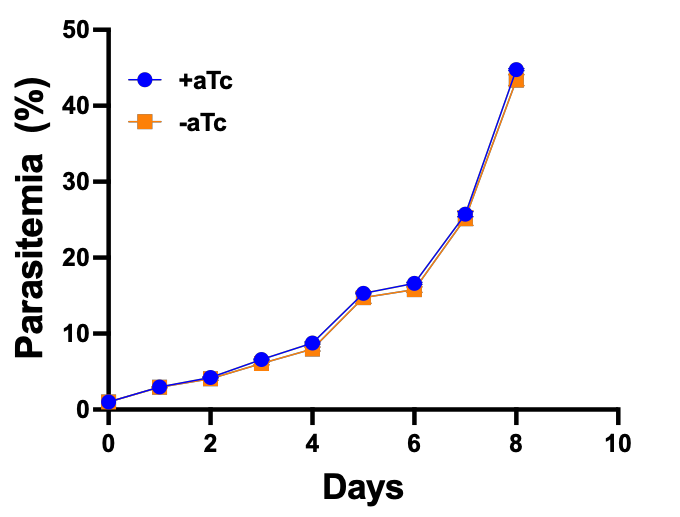
**(C)**  **(D)**


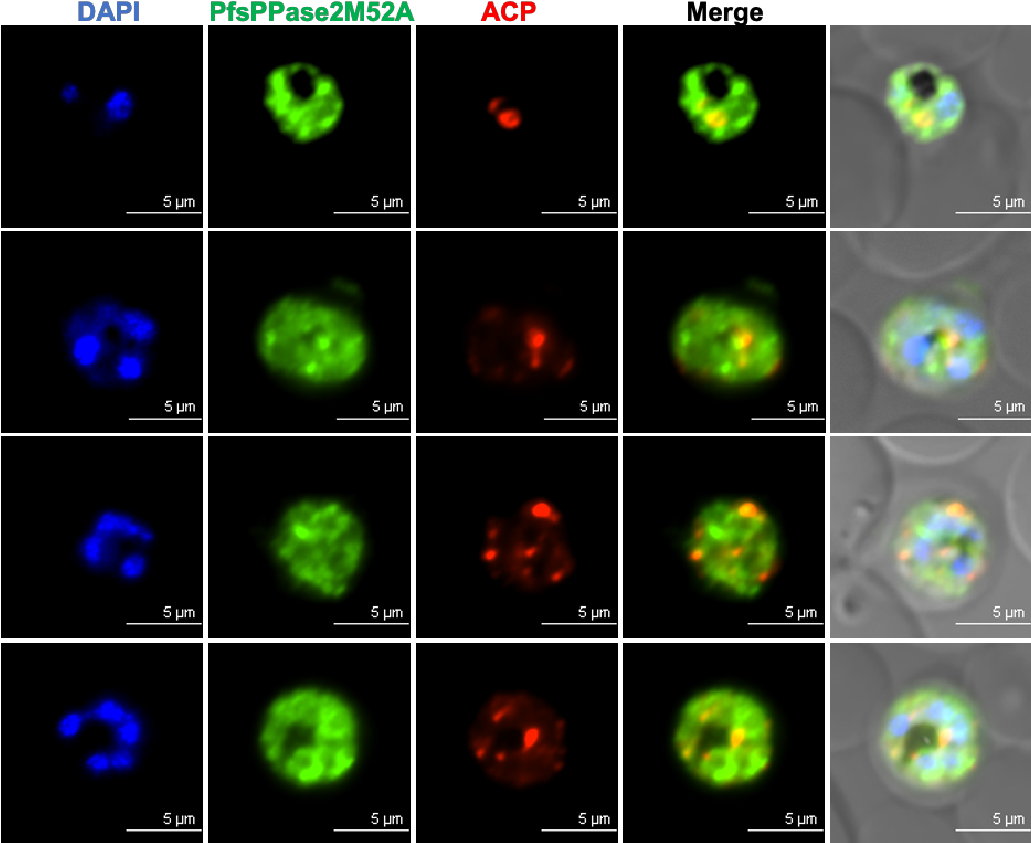


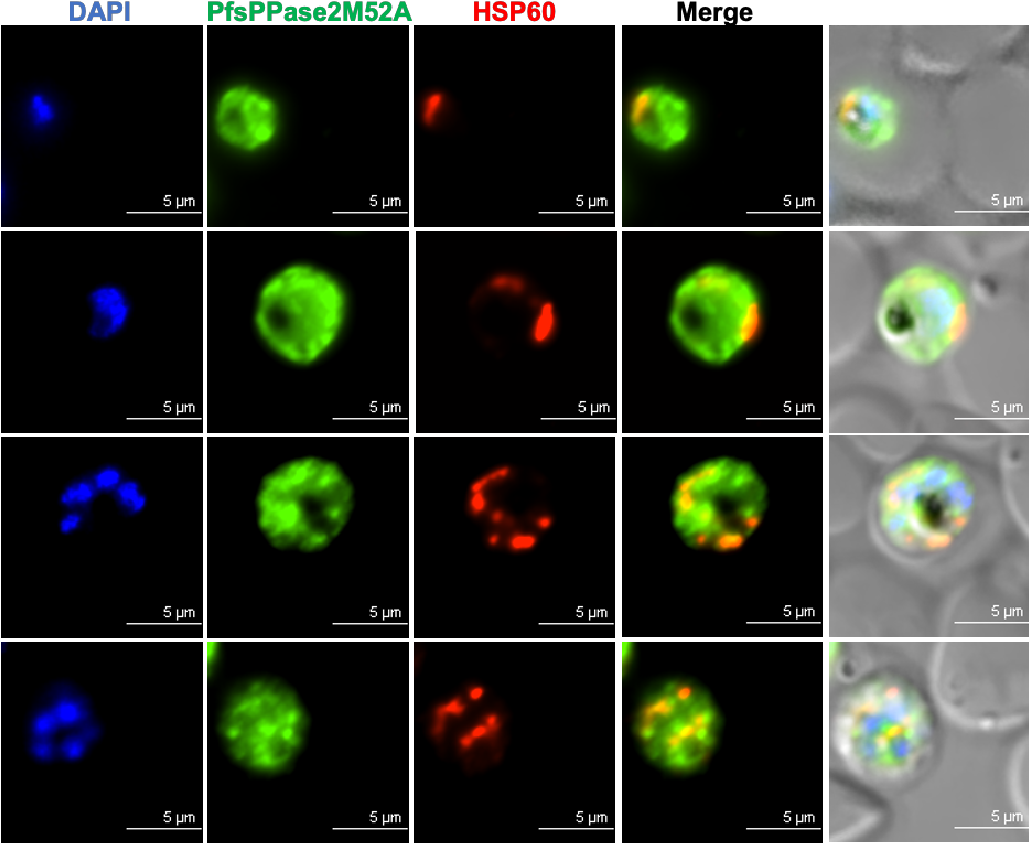
**(E) (F)**

**Supplementary Figure 11. PfsPPase2M52A mutant behaves the same as PfsPPase2.**

A, Western blot analysis of protein lysate from NF54attB-PfsPPase-3HA^APT^-PfsPPase2M52A-3Myc. The blot was probed with anti-HA to verify knockdown of the endogenous PfsPPases-3HA or with anti-Myc to show episomal expression of the M52A mutant. The blot was re-probed to show loading controls (anti-Exp2). B, Raw western blot for A. C, Giemsa-stained thin blood smears showing parasite morphological changes over 8 days after aTc removal. D, Parasitemia of the knockdown experiment determined by microscopic counting in triplicates. E, Colocalization of PfsPPase2M52A with the mitochondrion detected by anti-PfHSP60 (red). Scale bar, 5 µm. Pearson correlation coefficient between green and red fluorescence calculated from 12 parasites (0.8373 **±** 0.0614). F, Colocalization of PfsPPase2M52A with the apicoplast detected by anti-PfACP (red). Scale bar, 5 µm. Pearson correlation coefficient between green and red fluorescence from 12 parasites (0.8373 **±** 0.0790). Experiments A-D were repeated two times**.**

**References**

1. Ghorbal M, Gorman M, Macpherson CR, Martins RM, Scherf A, Lopez-Rubio JJ. 2014. Genome editing in the human malaria parasite Plasmodium falciparum using the CRISPR-Cas9 system. Nat Biotechnol 32:819-21.

2. Wagner JC, Platt RJ, Goldfless SJ, Zhang F, Niles JC. 2014. Efficient CRISPR-Cas9-mediated genome editing in Plasmodium falciparum. Nat Methods 11:915-8.

3. Hilgarth RS, Lanigan TM. 2020. Optimization of overlap extension PCR for efficient transgene construction. MethodsX 7:100759.

4. Ling L, Mulaka M, Munro J, Dass S, Mather MW, Riscoe MK, Llinás M, Zhou J, Ke H. 2020. Genetic ablation of the mitoribosome in the malaria parasite Plasmodium falciparum sensitizes it to antimalarials that target mitochondrial functions. Journal of Biological Chemistry 295:7235-7248.

5. Solebo O, Ling L, Nwankwo I, Zhou J, Fu TM, Ke H. 2023. Plasmodium falciparum utilizes pyrophosphate to fuel an essential proton pump in the ring stage and the transition to trophozoite stage. PLoS Pathog 19:e1011818.

6. Nina PB, Morrisey JM, Ganesan SM, Ke H, Pershing AM, Mather MW, Vaidya AB. 2011. ATP synthase complex of Plasmodium falciparum: dimeric assembly in mitochondrial membranes and resistance to genetic disruption. J Biol Chem 286:41312-41322.

7. Ke H, Dass S, Morrisey JM, Mather MW, Vaidya AB. 2018. The mitochondrial ribosomal protein L13 is critical for the structural and functional integrity of the mitochondrion in Plasmodium falciparum. Journal of Biological Chemistry 293:8128-8137.

8. Morano AA, Xu W, Navarro FM, Shadija N, Dvorin JD, Ke H. 2025. The dynamin-related protein PfDyn2 is essential for both apicoplast and mitochondrial fission in Plasmodium falciparum. mBio 16:e0303624.
